# SMAD4 depletion contributes to endocrine resistance by integrating ER and ERBB signaling in HR + HER2− breast cancer

**DOI:** 10.1038/s41419-024-06838-9

**Published:** 2024-06-24

**Authors:** Kang Li, Dan Shu, Han Li, Ailin Lan, Wenjie Zhang, Zhaofu Tan, Man Huang, Maria Lauda Tomasi, Aishun Jin, Haochen Yu, Meiying Shen, Shengchun Liu

**Affiliations:** 1 Department of Breast and Thyroid Surgery, The First Affiliated Hospital of Chongqing Medical University, 400016 Chongqing, China; 2 Department of Dermatology and Venereology, The First Affiliated Hospital of Chongqing Medical University, 400016 Chongqing, China; 3 Department of Breast Center, Chongqing University Three Gorges Hospital, Wanzhou, 404000 Chongqing, China; 4 Department of Medicine, Cedars-Sinai Medical Center, DAVIS Research Building 3096A, 8700 Beverly Blv, Los Angeles, CA 90048 USA; 5 Department of Immunology, School of Basic Medical Sciences, Chongqing Medical University, 400010 Chongqing, China

**Keywords:** Breast cancer, Cancer therapeutic resistance

## Abstract

Endocrine resistance poses a significant clinical challenge for patients with hormone receptor-positive and human epithelial growth factor receptor 2-negative (HR + HER2−) breast cancer. Dysregulation of estrogen receptor (ER) and ERBB signaling pathways is implicated in resistance development; however, the integration of these pathways remains unclear. While SMAD4 is known to play diverse roles in tumorigenesis, its involvement in endocrine resistance is poorly understood. Here, we investigate the role of SMAD4 in acquired endocrine resistance in HR + HER2− breast cancer. Genome-wide CRISPR screening identifies SMAD4 as a regulator of 4-hydroxytamoxifen (OHT) sensitivity in T47D cells. Clinical data analysis reveals downregulated SMAD4 expression in breast cancer tissues, correlating with poor prognosis. Following endocrine therapy, SMAD4 expression is further suppressed. Functional studies demonstrate that SMAD4 depletion induces endocrine resistance in vitro and in vivo by enhancing ER and ERBB signaling. Concomitant inhibition of ER and ERBB signaling leads to aberrant autophagy activation. Simultaneous inhibition of ER, ERBB, and autophagy pathways synergistically impacts SMAD4-depleted cells. Our findings unveil a mechanism whereby endocrine therapy-induced SMAD4 downregulation drives acquired resistance by integrating ER and ERBB signaling and suggest a rational treatment strategy for endocrine-resistant HR + HER2− breast cancer patients.

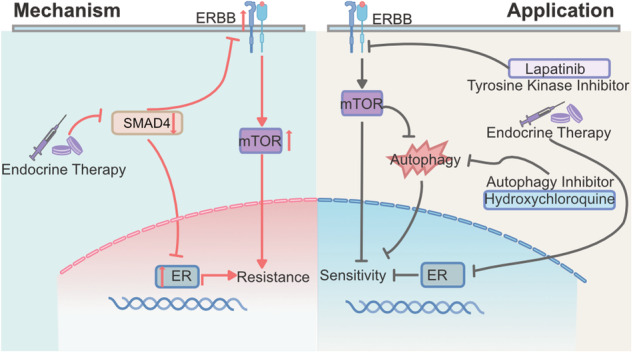

## Introduction

Worldwide, breast cancer is the most commonly diagnosed malignancy in women [1]. Despite advances in patient management, breast cancer remains the second leading cause of cancer-related death [2]. It is estimated that 75% of breast cancers are hormone receptor positive (HR+) [3]. The ER is an estradiol (E2)-activated transcription factor [4] that promotes the initiation, progression, and metastasis of HR + HER2− tumors. As a result, endocrine therapies targeting the ER pathway, such as tamoxifen (TAM) and fulvestrant (FVE), are the mainstay of treatment for both early and advanced breast cancer [5]. Despite an initial response responding to endocrine therapy, more than half of patients eventually develop resistance to treatment over time [6, 7]. This represents a significant clinical challenge to the success of HR + HER2− breast cancer treatment [8]. Therefore, there is an urgent need to investigate the underlying molecular mechanisms and identify novel targets to overcome this clinical challenge. The development of acquired endocrine resistance has been attributed to aberrant activation of the ER and ERBB pathways [9, 10]. However, it remains unclear how these pathways are activated and coordinated to work together. Understanding the mechanisms involved will facilitate the development of predictive, diagnostic, and therapeutic options for patients with acquired endocrine resistance.

SMAD4 functions as a common signal transducer in the transforming growth factor (TGF) superfamily [11]. SMAD4 has been extensively studied in pancreatic cancer and colorectal cancer due to its high mutation rate [12, 13]. A small percentage of invasive ductal carcinomas have homozygous deletions of SMAD4 [14, 15]. Despite the low mutation rate of SMAD4 in breast cancer, loss of SMAD4 is associated with a poor prognosis [16]. In HR + HER2− breast cancer cells, SMAD4 has been shown to act as an ER transcriptional corepressor [17] and to inhibit tumor growth by inducing apoptosis [18].

In this study, we investigated the role of SMAD4 in the development of resistance to endocrine therapy and the underlying mechanisms. We showed that SMAD4 downregulation induced by endocrine therapy leads to acquired endocrine resistance by integrating ER and ERBB signaling. Simultaneous inhibition of ER and ERBB signaling led to aberrant activation of autophagy. Thus, simultaneous targeting of ER activity, ERBB signaling, and autophagy may be beneficial in overcoming acquired resistance to endocrine therapy.

## Results

### CRISPR screening identifies SMAD4 as a gene involved in endocrine resistance

**Fig. 1:**
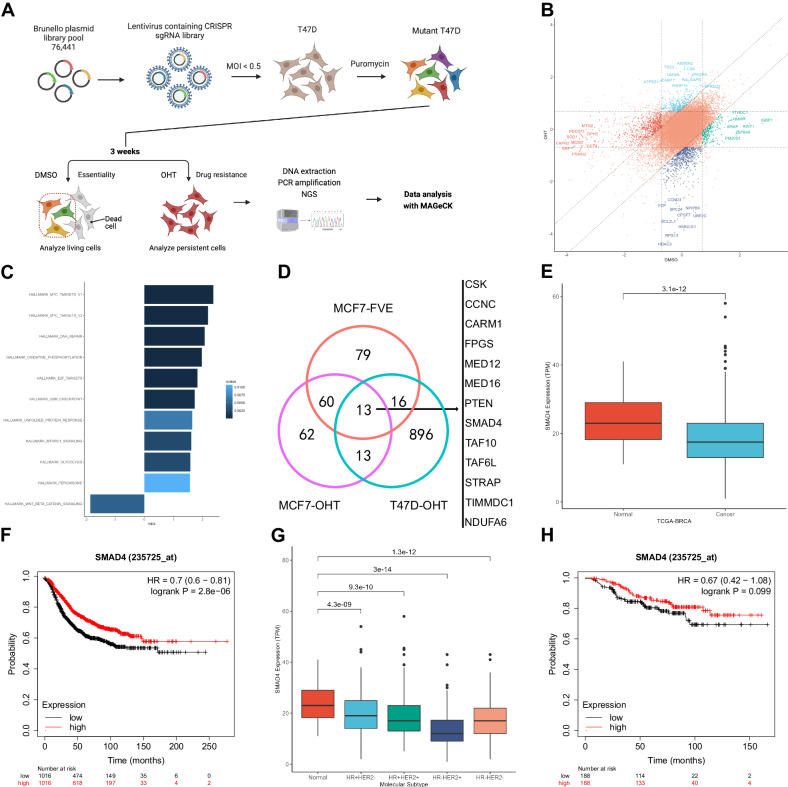
CRISPR screening identifies SMAD4 as a gene involved in endocrine resistance. **A** Workflow for genome-wide CRISPR screening using OHT. MOI, multiplicity of infection. NGS, next-generation sequencing. **B** Nine-square dot plot displaying gene enrichment differences calculated by the MAGeCK MLE algorithm in CRISPR screening with OHT. **C** GSEA bar plot revealing enriched MSigDB gene sets. Bar width corresponds to the normalized enrichment score (NES), while bar colors indicate the *q*-value. **D** Venn diagram illustrating the number of differentially enriched genes in the OHT screen of MCF7 cells (MCF7-OHT), the FVE screen of MCF7 cells (MCF7-FVE), and the OHT screen of T47D cells (T47D-OHT). T47D-OHT represents our CRISPR screening results, while MCF7-OHT and MCF7-FVE data are from GSE123283. An absolute enrichment difference >1 was used for result filtering. Candidate targets overlapping in the three groups are listed on the right. **E** Box plots showing SMAD4 expression differences between normal (*n* = 102) and cancer (*n* = 1022) tissues in the TCGA breast cancer (TCGA-BRCA) dataset. Expression data were normalized to transcripts per kilobase million (TPM) values. *p*-values (Student’s *t* test) are indicated. The median line, box borders (25th and 75th percentiles), and whiskers (1.5 times the interquartile range) are shown. **F** Survival analysis in K-M Plotter: Low SMAD4 expression correlates with poor prognosis. **G** Box plots displaying SMAD4 expression differences among normal (*n* = 102), HR + HER2− (*n* = 486), HR + HER2+ (*n* = 269), HR-HER2+ (*n* = 76), and HR-HER2− (*n* = 191) cancers in the TCGA-BRCA dataset. Expression data were normalized to transcripts per kilobase million (TPM) values. *p*-values (Student’s *t* test) are indicated. The median line, box borders (25th and 75th percentiles), and whiskers (1.5 times the interquartile range) are shown. **H** Survival analysis in K-M Plotter: Low SMAD4 expression correlates with poor outcome on endocrine therapy.

To better characterize the molecular features of endocrine resistance, we performed a CRISPR screening using the Brunello library in OHT-treated T47D cells (Fig. 1A and Supplementary Table 1). The asynchronous cells used for this screen were cultured in estrogen-rich media and exposed to 1 μM OHT for a total of 3 weeks. Enrichment difference analysis of the genes showing changes after OHT treatment revealed known breast cancer oncogenes such as HDAC3, UBE2C and CPSF7 among the depleted genes and reported breast cancer suppressors such as CSK, SPRED2 and TSC2 among the enriched genes (Fig. 1B). We then performed gene set enrichment analysis (GSEA) using GO, KEGG, and MSigDB Hallmark gene sets to investigate the underlying mechanisms of endocrine resistance. Several processes and pathways, such as cell cycle regulation, oxidative phosphorylation and the mTOR pathway, were enriched and known to be potential contributors to endocrine resistance (Fig. 1C and Supplementary Fig. 1A, B). To identify targets, we compared our dataset with GSE123283 [19], a CRISPR screening dataset obtained from the OHT and FVE treatment of MCF7 cells. Thirteen reliable candidate targets were obtained after intersecting our differentially enriched genes with those of GSE123283 (Fig. 1D). Among these candidate targets, CSK [20], CARM1 [21], MED16 [22] and PTEN [23] had been reported in endocrine resistance, further demonstrating the reliability of these candidate genes. To further filter candidate targets, we analyzed the TCGA [24] RNA-seq data for expression difference and the Kaplan–Meier Plotter online database for survival difference (Supplementary file 1) [25]. Only SMAD4 showed lower expression in cancer tissues and was simultaneously associated with poor prognosis (Fig. 1E, F). Most importantly, we found that low SMAD4 expression indicated a poorer response to the effect of endocrine therapy in HR+ patients (Fig. 1G, H). Finally, we selected SMAD4 for the next study.

### Downregulation of SMAD4 contributes to endocrine resistance

**Fig. 2:**
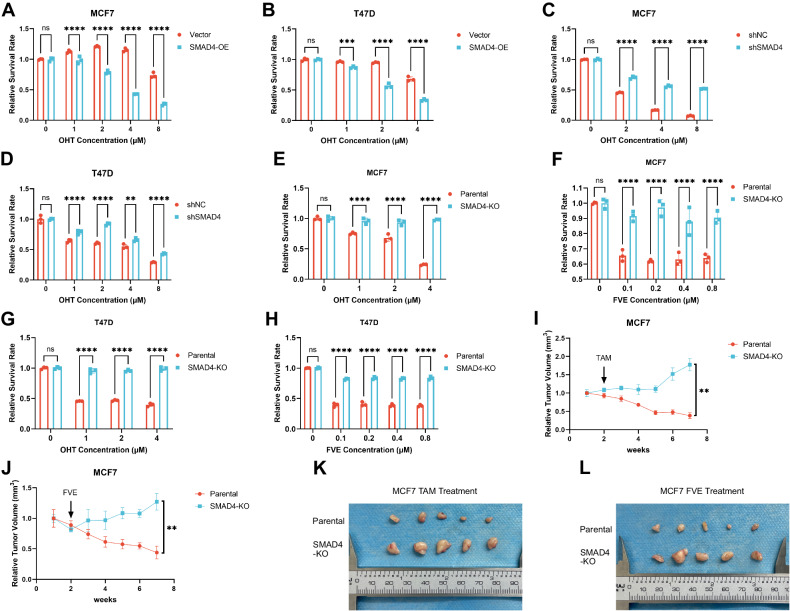
Downregulation of SMAD4 contributes to endocrine resistance. **A**, **B** CCK8 assay conducted in SMAD4 overexpressing (SMAD4-OE) and control (Vector) MCF7/T47D cells treated with OHT for 72 h. Error bars represent the mean ± SEM of *n* = 3 biological replicates. Statistical significance denoted by asterisks (*p < 0.05, **p < 0.01, ***p < 0.001, ****p < 0.0001, two-way ANOVA). **C**, **D** CCK8 assay performed in SMAD4 knockdown (shSMAD4) and control (shNC) MCF7/T47D cells treated with OHT for 72 h. Error bars represent the mean ± SEM of *n* = 3 biological replicates. Statistical significance indicated by asterisks (*p < 0.05, **p < 0.01, ***p < 0.001, ****p < 0.0001, two-way ANOVA). **E**–**H** CCK8 assay carried out in SMAD4-KO and parental MCF7/T47D cells treated with OHT/FVE for 72 h. Error bars represent the mean ± SEM of *n* = 3 biological replicates. Statistical significance denoted by asterisks (*p < 0.05, **p < 0.01, ***p < 0.001, ****p < 0.0001, two-way ANOVA). **I**, **J** Line graphs depicting relative tumor size over time with OHT/FVE treatment. Error bars represent mean ± SEM of *n* = 5 biological replicates. Statistical significance indicated by *p*-value (Mann–Whitney *U* test). **K**, **L** Photos display tumor samples excised at the end of the experiment.

To evaluate the role of SMAD4 in acquired endocrine resistance, we performed gain-of-function and loss-of-function assays. For the gain-of-function assays, we constructed SMAD4-overexpressing MCF7 and T47D breast cancer cells (Supplementary Fig. 2A–C and original data). After treatment with different concentrations of OHT, CCK8 assays revealed that SMAD4 overexpression dramatically reduced the viability of MCF7 and T47D cells in a dose-dependent manner (Fig. 2A, B). In the loss-of-function assays, MCF7 and T47D were selected for stable knockdown of SMAD4 (Supplementary Fig. 2D, E and original data). The viability of the SMAD4-knockdown MCF7 and T47D cells was higher than that of the control cells after treatment with different doses of OHT (Fig. 2C, D). SMAD4-knockout strains were further developed using MCF7 and T47D cell lines (Supplementary Fig. 2F and original data). CCK8 assays showed that the viability of SMAD4-knockout MCF7 and T47D cells was significantly increased compared to that of parental cells (Fig. 2E–H). Colony formation assays also indicated that SMAD4-knockout MCF7 and T47D cells were resistant to OHT (Supplementary Fig. 2G, H). Furthermore, we performed xenograft animal experiments to verify the role of SMAD4 in the development of acquired endocrine resistance. Consistently, tumor size and weight dramatically increased in the SMAD4-KO group but decreased in the control group after treatment with TAM or FVE for 6 weeks. (Fig. 2I–L, Supplementary Fig. 2I–J). Taken together, our results suggest that SMAD4 depletion is closely associated with endocrine resistance.

### Endocrine therapy-induced SMAD4 downregulation is independent of ER signaling

**Fig. 3:**
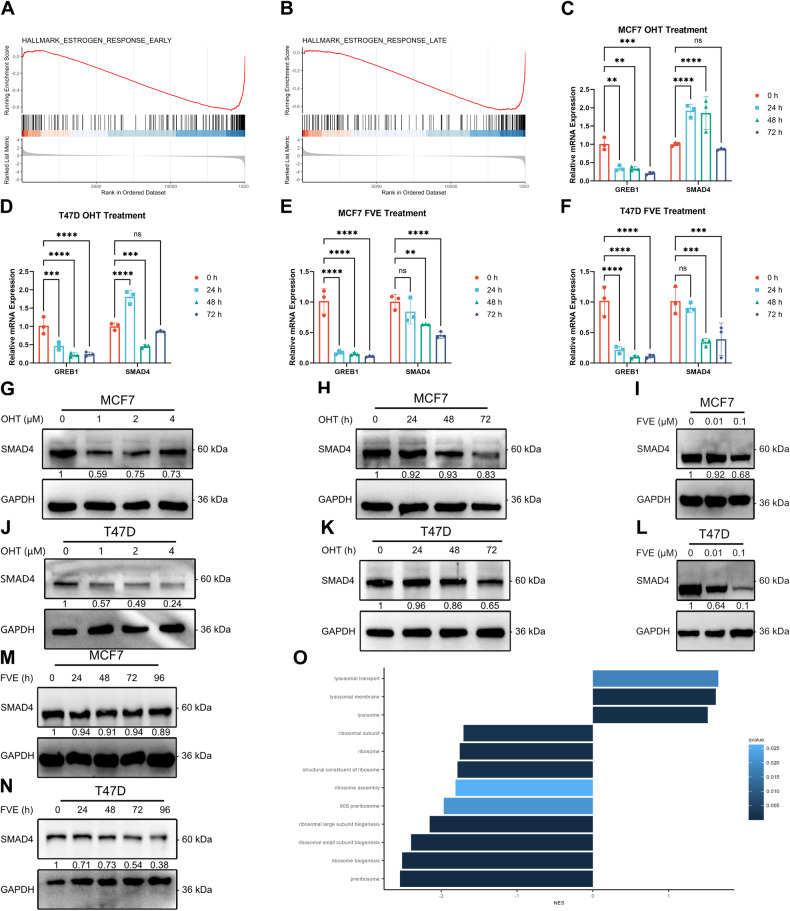
Endocrine therapy-induced SMAD4 downregulation is independent of ER signaling. **A**, **B** GSEA enrichment plots illustrating enriched gene sets in OHT-treated MCF7 cells compared to DMSO-treated cells. A red horizontal bar transitioning to blue indicates a shift from positively correlated genes (red) to negatively correlated genes (blue). **C**–**F** RT-qPCR analysis of GREB1 and SMAD4 mRNA expression in MCF7/T47D cells after treatment with 1 μM OHT or 0.1 μM FVE for 0, 24, 48, and 72 h. Error bars represent the mean ± SEM of *n* = 3 biological replicates. Statistical significance indicated by asterisks (*p < 0.05, **p < 0.01, ***p < 0.001, ****p < 0.0001, two-way ANOVA). **G** Immunoblot assay depicting protein expression of SMAD4 in MCF7 cells treated with OHT (0, 1, 2, 4 μM) for 24 h. GAPDH utilized as a loading control. **H** Immunoblot assay illustrating protein expression of SMAD4 in MCF7 cells treated with 1 μM OHT for 0, 24, 48, and 72 h. GAPDH employed as loading control. **I** Immunoblot assay showing protein expression of SMAD4 in MCF7 cells treated with FVE (0, 0.01, 0.1 μM) for 24 h. GAPDH used as loading control. **J** Immunoblot assay demonstrating protein expression of SMAD4 in T47D cells treated with OHT (0, 1, 2, 4 μM) for 24 h. GAPDH employed as loading control. **K** Immunoblot assay depicting protein expression of SMAD4 in T47D cells treated with 1 μM OHT for 0, 24, 48, and 72 h. GAPDH utilized as loading control. **L** Immunoblot assay illustrating protein expression of SMAD4 in T47D cells treated with FVE (0, 0.01, 0.1 μM) for 24 h. GAPDH employed as loading control. **M** Immunoblot assay showing protein expression of SMAD4 in MCF7 cells treated with 0.1 μM FVE for 0, 24, 48, 72, and 96 h. GAPDH utilized as loading control. **N** Immunoblot assay demonstrating protein expression of SMAD4 in T47D cells treated with 0.1 μM FVE for 0, 24, 48, 72, and 96 h. GAPDH employed as loading control. **O** Bar graph of GSEA showing enriched GO gene sets. Bar width corresponds to the normalized enrichment score (NES), while bar colors indicate the q-value.

To investigate the relationship between SMAD4 expression and endocrine therapy, we first performed RNA-seq on OHT-treated MCF7 cells (Supplementary Fig. 3A and Supplementary Table 2). GSEA revealed that the main inhibited signaling is ER (Fig. 3A, B). The differential expression result of ER target gene GREB1 showed significant inhibition by OHT, while SMAD4 didn’t show statistically significant downregulation (Supplementary Fig. 3B). To determine whether ER regulates SMAD4 expression, we evaluated the expression of GREB1 and SMAD4 in MCF7 and T47D cells upon OHT or FVE treatment. RT-qPCR results showed that OHT or FVE could strongly inhibit GREB1, but the expression of SMAD4 upon OHT and FVE treatment didn’t show consistent inhibition (Fig. 3C–F). These results suggest that endocrine therapy couldn’t transcriptionally downregulate SMAD4 by inhibiting ER signaling. To determine whether SMAD4 expression is altered with the development of endocrine resistance, we next evaluated SMAD4 expression at the protein level by immunoblot analysis in MCF7 and T47D cells after treatment with OHT or FVE using a concentration or time gradient approach. The results showed that SMAD4 downregulation was time- and concentration-dependent (Fig. 3G–N and original data). GSEA with GO gene sets suggested that inhibited ribosome function and activated lysosome function may contribute to the regulation of SMAD4 expression upon endocrine therapy (Fig. 3O). Taken together, these results suggest that endocrine therapy contributes to the downregulation of SMAD4 protein, which may contribute to the emergence of endocrine resistance.

### Activation of ER signaling due to SMAD4 depletion is not the sole factor contributing to endocrine resistance

**Fig. 4:**
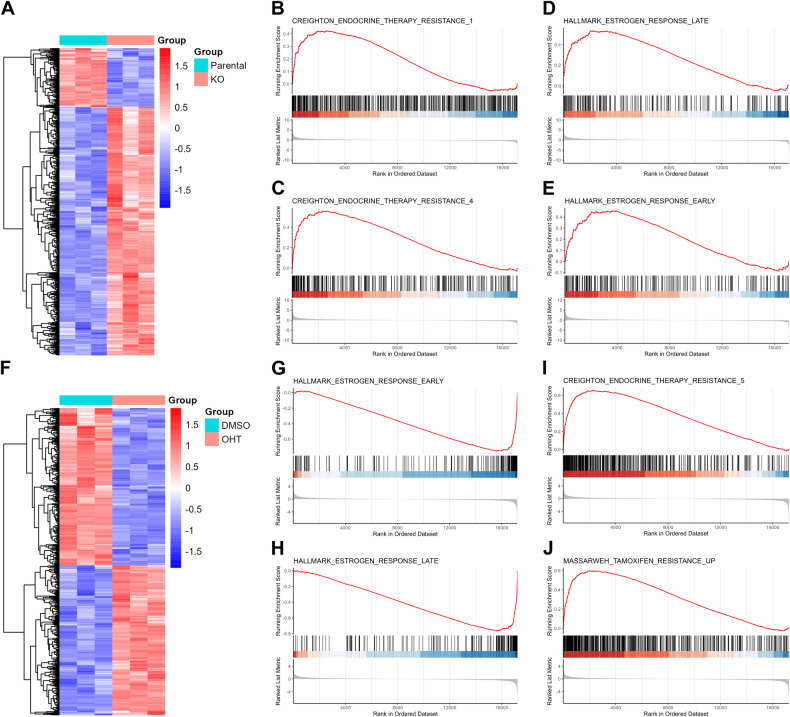
Activation of ER signaling due to SMAD4 depletion is not the sole factor contributing to endocrine resistance. **A** Heatmap displaying differential gene expression in parental vs. SMAD4 knockout (KO) MCF7 cells (absolute log2-fold change > 1, Benjamini-Hochberg adjusted *p* < 0.05). Among these genes, 700 exhibited significant upregulation, while 166 genes showed significant downregulation in the SMAD4 KO group. **B**–**E** GSEA enrichment plots demonstrating enriched gene sets in SMAD4 KO MCF7 cells compared to parental cells. A red horizontal bar transitioning to blue signifies a shift from positively correlated genes (red) to negatively correlated genes (blue). **F** Heatmap depicting differential gene expression between DMSO (DMSO) and OHT (OHT) treatment groups in SMAD4-depleted MCF7 cells (absolute log2-fold change > 1, Benjamini-Hochberg adjusted *p* < 0.05). Among these genes, 305 displayed significant upregulation, while 320 genes showed significant downregulation in the OHT group. **G**–**J** GSEA enrichment plots illustrating enriched gene sets in SMAD4 KO MCF7 cells treated with OHT compared to SMAD4 KO MCF7 cells treated with DMSO. A red horizontal bar transitioning to blue indicates a shift from positively correlated genes (red) to negatively correlated genes (blue).

To investigate the potential mechanisms of endocrine therapy resistance due to SMAD4 depletion, we performed RNA-seq to examine the transcriptional profile of SMAD4-depleted MCF7 cells (Fig. 4A and Supplementary Table 3). Using endocrine resistance-related gene sets from MSigDB, GSEA revealed that endocrine resistance-related gene sets were enriched in SMAD4-depleted MCF7 cells compared to the parental MCF7 cells (Fig. 4B, C). These results further confirmed that SMAD4 loss is an effective contributor to endocrine resistance. Since endocrine resistance has long been associated with abnormal activation of the ER pathway [9], and previous studies have shown that SMAD4 acts as a corepressor of the ER [17]. We further investigated whether SMAD4 depletion could activate ER signaling at the transcriptome level. GSEA with hallmark gene sets from MSigDB revealed that estrogen response-related gene sets were enriched in MCF7 SMAD4-KO cells compared to MCF7 parental cells (Fig. 4D, E), suggesting that SMAD4 depletion led to abnormal ER pathway activation. To validate whether abnormal ER signaling was the main factor leading to endocrine therapy, we next performed a transcriptome rescue assay by treating MCF7 SMAD4-KO cells with OHT (Fig. 4F and Supplementary Table 4). GSEA with hallmark gene sets from MSigDB revealed that ER signaling activated by SMAD4 loss was inhibited by OHT (Fig. 4G, H). And GSEA with endocrine resistance-related gene sets from MSigDB revealed that endocrine resistance-related gene sets were activated in MCF7 SMAD4-KO cells upon OHT treatment (Fig. 4I, J and Supplementary Fig. 4A). We further validated these results in MCF7 SMAD4 knockdown and knockout cells by quantifying GREB1 and/or TFF1 using RT-qPCR, and the results showed that GREB1 and TFF1 were upregulated upon SMAD4 knockdown/knockout, but their upregulation was again suppressed by OHT (Supplementary Fig. 4B, C). These results indicate that the loss of SMAD4 contributes to the activation of ER signaling, but the activation of ER signaling is not the only factor underlying SMAD4 depletion-induced endocrine resistance.

### SMAD4 depletion activates mTOR signaling, contributing to endocrine resistance

**Fig. 5:**
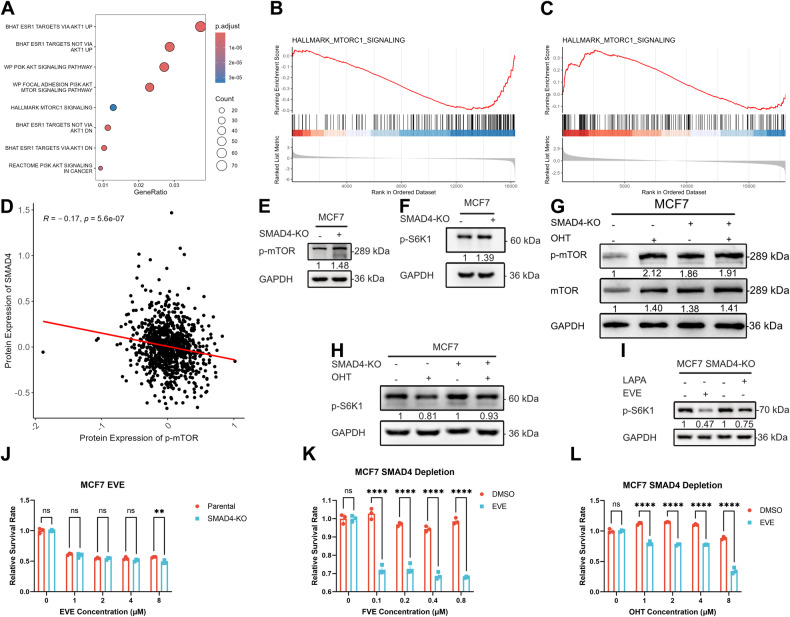
SMAD4 depletion activates mTOR signaling, contributing to endocrine resistance. **A** Dot plot displaying overrepresented MSigDB gene sets in SMAD4 knockout MCF7 cells compared to parental cells. Dot sizes indicate gene number, while dot colors represent adjusted *p*-values (p.adjust). **B** GSEA enrichment plots revealing enriched gene sets in OHT-treated MCF7 cells compared to DMSO-treated MCF7 cells. A red horizontal bar transitioning to blue indicates a shift from positively correlated genes (red) to negatively correlated genes (blue). **C** GSEA enrichment plots demonstrating enriched gene sets in T47D cells post-OHT-CRISPR screening. A red horizontal bar transitioning to blue indicates a shift from positively correlated genes (red) to negatively correlated genes (blue). **D** Dot plot illustrating protein expression correlation between SMAD4 and p-mTOR, with correlation coefficient and *p*-value provided. **E**, **F** Immunoblot analysis of p-mTOR and *p*-S6K1 protein expression in SMAD4 knockout and parental MCF7 cells. GAPDH served as a loading control. **G**, **H** Immunoblot analysis of p-mTOR/mTOR and p-S6K1 protein expression in SMAD4 knockout and parental MCF7 cells treated with 1 μM OHT. GAPDH was utilized as a loading control. **I** Immunoblot analysis of p-S6K1 protein expression in SMAD4 knockout MCF7 cells following treatment with DMSO, EVE, or LAPA. GAPDH served as a loading control. **J** CCK8 assays conducted in parental and SMAD4-knockout MCF7 cells treated with EVE for 72 h. Error bars represent mean ± SEM of *n* = 3 biological replicates. Statistical significance is indicated by asterisks (*p < 0.05, **p < 0.01, ***p < 0.001, ****p < 0.0001, two-way ANOVA). **K**, **L** CCK8 assays performed in SMAD4-knockout MCF7 cells treated with OHT or FVE alone or in combination with 1 μM EVE for 72 h. Error bars represent mean ± SEM of *n* = 3 biological replicates. Statistical significance denoted by asterisks (*p < 0.05, **p < 0.01, ***p < 0.001, ****p < 0.0001, two-way ANOVA).

Previous studies have reported that ER signaling can be activated by the mTOR pathway through ER phosphorylation [26]. Our over-representation analysis (ORA) with gene sets from MSigDB revealed that a proportion of ER target genes were associated with the mTOR pathway (Fig. 5A), suggesting that mTOR signaling may contribute to the activation of ER signaling upon SMAD4 depletion. Furthermore, we found that mTOR signaling was repressed by OHT in MCF7 cells (Fig. 5B), suggesting that aberrant mTOR signaling may be responsible for endocrine resistance. Most importantly, our CRISPR screening revealed that activation of mTOR signaling contributes to endocrine resistance (Fig. 5C). All these results led us to investigate whether SMAD4 depletion contributes to aberrant activation of mTOR signaling. Our initial analysis of RPPA data from the CCLE cell line revealed a reverse correlation between SMAD4 expression and phosphorylated mTOR (p-mTOR) levels (Fig. 5D and Supplementary Fig. 5A). Subsequently, immunoblot assays were conducted to assess mTOR signaling markers in SMAD4-depleted MCF7 and T47D cells. We observed a significant increase in both p-mTOR and phosphorylated S6K1 (p-S6K1) protein levels compared to parental cells (Fig. 5E, F and original data), confirming that the loss of SMAD4 leads to mTOR signaling activation. Additionally, we found that 4-hydroxytamoxifen (OHT) only marginally suppressed mTOR signaling in SMAD4-depleted cells compared to parental cells (Fig. 5G, H and original data). These findings suggest that mTOR activation contributes to endocrine resistance. Next, we used the mTOR allosteric inhibitor everolimus (EVE) to perform rescue experiments. First, we confirmed the inhibition efficiency of the mTOR inhibitor EVE on mTOR signaling by immunoblotting (Fig. 5I and original data), and then performed cell viability assay with EVE in MCF7 parental and SMAD4-KO cells, the similar inhibition rate between MCF7 parental and SMAD4-KO cells suggests that activation of mTOR signaling alone could not lead to endocrine resistance (Fig. 5J). Finally, we performed phenotypic rescue assays in SMAD4-KO MCF7 cells with EVE to verify the contribution of the mTOR pathway to endocrine resistance. The results showed significantly reduced cell viability in the EVE rescue group compared to the OHT or FVE alone group (Fig. 5K, L). These results suggest that SMAD4 depletion induces endocrine resistance by simultaneously increasing ER and mTOR signaling.

### ERBB signaling contributes to the activation of mTOR signaling

**Fig. 6:**
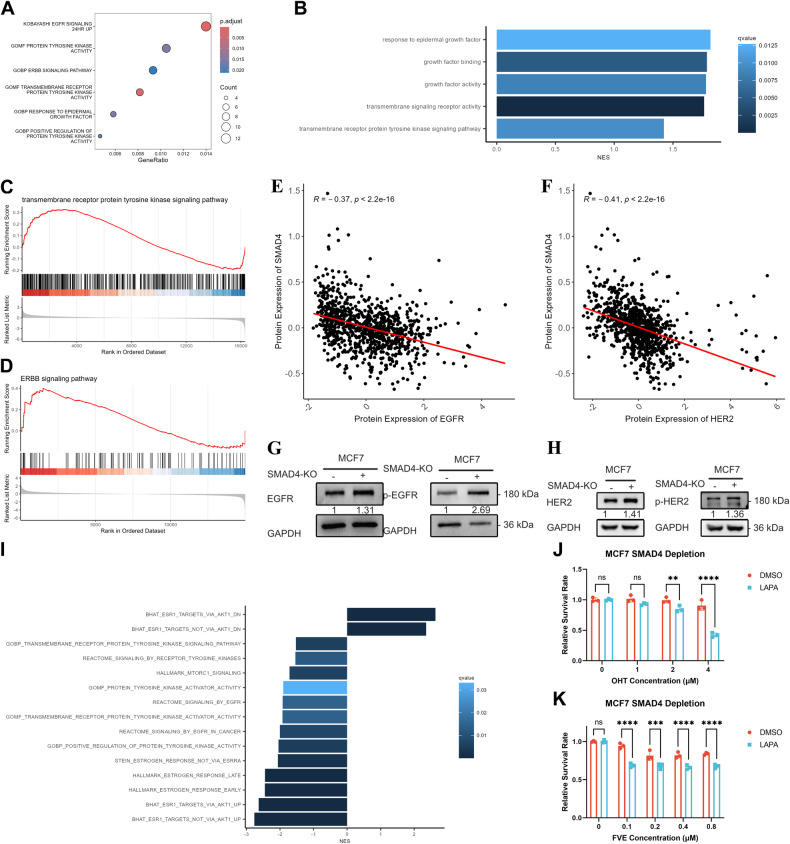
ERBB signaling contributes to mTOR signaling activation. **A** The dot plot displays enriched gene sets in SMAD4 knockout MCF7 cells compared to parental cells. Dot size is scaled by the number of genes, while dot color represents the adjusted *p*-value (*p*.adjust). **B** Bar graph of GSEA illustrating enriched MSigDB gene sets. Bar width corresponds to the normalized enrichment score (NES), while bar colors indicate the *q*-value. **C**, **D** GSEA enrichment plots showing enriched gene sets in OHT-treated MCF7 cells compared to DMSO-treated MCF7 cells. A red horizontal bar transitioning to blue indicates a change from positively correlated genes (red) to negatively correlated genes (blue). **E**, **F** The dot plot demonstrates protein expression correlation between SMAD4 and EGFR/HER2, with correlation coefficient and *p*-value indicated. **G** Immunoblot analysis of EGFR and p-EGFR protein expression in SMAD4 knockout and parental MCF7 cells. GAPDH employed as loading control. **H** Immunoblot analysis of HER2 and p-HER2 protein expression in SMAD4 knockout and parental MCF7 cells. GAPDH used as loading control. **I** Bar graph of GSEA depicting enriched MSigDB gene sets in SMAD4 knockout MCF7 cells treated with hydroxytamoxifen and lapatinib. Dot size is scaled by the number of genes, and dot color indicates the adjusted *p*-value (*p*.adjust). **J**, **K** CCK8 assays performed in SMAD4 knockout MCF7 cells treated with 4-hydroxytamoxifen (OHT) or fulvestrant (FVE) alone or in combination with 1 μM lapatinib (LAPA), for 72 h. Error bars represent the mean ± SEM of *n* = 3 biological replicates. Statistical significance denoted by asterisks (*p < 0.05, **p < 0.01, ***p < 0.001, ****p < 0.0001, two-way ANOVA).

To determine which factor mediates the activation of mTOR signaling upon SMAD4 depletion, we first searched the DEGs between MCF7 parental and SMAD4 KO cells. None of the DEGs caught our attention because there are no clinical drugs readily available for these genes. The activation of mTOR signaling in endocrine resistance frequently implicates overexpression of receptor tyrosine kinases (RTKs), including epidermal growth factor receptor (EGFR) and HER2, which participate in pathways collectively known as growth factor or ERBB signaling [27, 28]. We then searched the ORA results and found that MSigDB terms related to ERBB signaling and protein tyrosine kinase activity were overrepresented in our DEGs (Fig. 6A). Similarly, growth factor and protein tyrosine kinase signaling-related gene sets from GO GSEA were activated (Fig. 6B). Most importantly, protein tyrosine kinase signaling and ERBB signaling were activated in OHT-treated MCF7 cells (Fig. 6C, D). These results suggest that activation of the mTOR pathway involves overexpressed RTKs [27, 28]. Subsequently, we investigated the relationship between SMAD4 and EGFR/HER2. Initial analysis of RPPA data from the CCLE cell line revealed a reverse correlation between SMAD4 and EGFR/HER2 expression (Fig. 6E, F). Immunoblotting was then employed to assess EGFR/HER2 expression and related phosphorylation levels in SMAD4 knockout and parental MCF7 cells. Our findings indicated that SMAD4 depletion increased EGFR/HER2 expression and phosphorylation (Fig. 6G, H and original data). Similarly, activation of ERK signaling was observed alongside elevated ERBB activity (Supplementary Fig. 5B and original data). Next, clinically available inhibitor lapatinib (LAPA) was used to perform rescue experiments. The inhibition efficiency of LAPA on ERBB signaling was validated by immunoblotting (Fig. 5I and original data). Then, we performed a transcriptome rescue assay by RNA-seq in SMAD4-depleted MCF7 cells. According to GSEA, combined OHT and LAPA treatment inhibited SMAD4 depletion-induced signaling, including that involving ERBB, and ER signaling (Fig. 6I). To further verify whether ERBB signaling activation contributes to SMAD4 depletion-driven endocrine resistance, we performed phenotypic rescue assays in SMAD4-KO MCF7 cells with LAPA. In SMAD4-KO cells, concurrent suppression of ER and ERBB signaling with OHT/FVE and LAPA resulted in a significant reduction in cell viability compared to treatment with OHT/FVE alone (Fig. 6J, K). Taken together, these results suggest that SMAD4 depletion-induced ERBB signaling contributes to endocrine resistance.

### Combined inhibition of ER and ERBB signaling resulted in aberrant autophagy activation

**Fig. 7:**
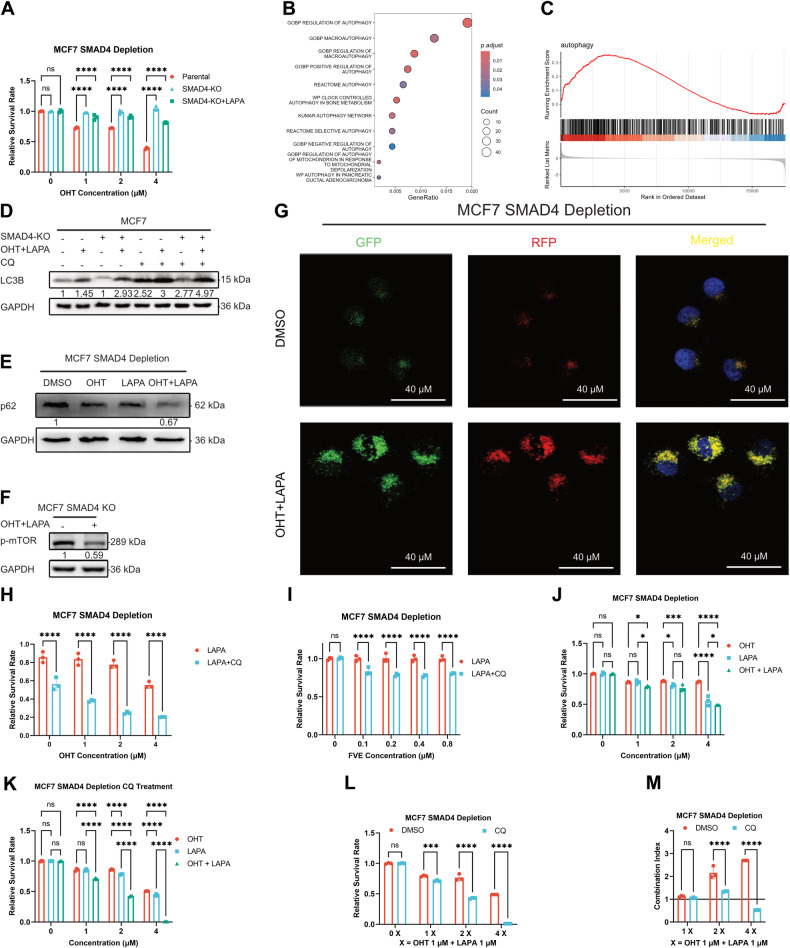
Combined inhibition of ER and ERBB signaling resulted in aberrant autophagy activation. **A** CCK8 assay conducted in MCF7 parental, SMAD4-KO, or 1 μM LAPA treated SMAD4-KO cells after treatment with OHT for 72 h. Error bars represent the mean ± SEM of *n* = 3 biological replicates. Statistical significance indicated by asterisks (*p < 0.05, **p < 0.01, ***p < 0.001, ****p < 0.0001, two-way ANOVA). **B** Dot plot displaying overrepresented MSigDB gene sets in OHT + LAPA treated SMAD4 knockout MCF7 cells compared to DMSO treated SMAD4 knockout cells. Dot size is scaled by the number of genes, while dot color represents the adjusted *p*-value (*p*.adjust). **C** GSEA enrichment plot demonstrating enriched gene sets in OHT- and LAPA-treated MCF7 SMAD4-KO cells compared to DMSO-treated cells. A red horizontal bar transitioning to blue indicates a change from positively correlated genes (red) to negatively correlated genes (blue). **D**–**F** Immunoblot analysis of LC3B, P62, and p-mTOR protein expression in SMAD4-depleted MCF7 cells treated with 1 μM OHT or 1 μM OHT + 1 μM LAPA. GAPDH employed as loading control. **G** Autophagic flux analyzed by mRFP-GFP-LC reporter via confocal microscopy. **H**, **I** CCK8 assays performed in SMAD4 knockout MCF7 cells treated with 4-hydroxytamoxifen (OHT) or fulvestrant (FVE) in combination with 1 μM lapatinib (LAPA) or 1 μM lapatinib (LAPA) plus 1 μM hydroxychloroquine (CQ) for 72 h. Error bars represent the mean ± SEM of *n* = 3 biological replicates. Statistical significance denoted by asterisks (*p < 0.05, **p < 0.01, ***p < 0.001, ****p < 0.0001, two-way ANOVA). **J**–**M** Synergy experiments with OHT and LAPA in SMAD4 knockout MCF7 cells. Error bars represent the mean ± SEM of *n* = 3 biological replicates. Statistical significance indicated by asterisks (*p < 0.05, **p < 0.01, ***p < 0.001, ****p < 0.0001, two-way ANOVA). **J** Cell viability of SMAD4-depleted MCF7 cells treated with OHT, LAPA, or OHT + LAPA. **K** Cell viability of cells treated as in J, but autophagy was simultaneously inhibited by 1 μM CQ. **L** Cell viability of cells treated with OHT plus LAPA in combination with or without 1 μM CQ. **M** Bar graph showing the combination indices. A Y-intercept of 1 classified the combined effect as additive (CI = 1), CI < 1 as synergistic, and CI > 1 as antagonistic.

Our results suggested that the combined inhibition of ER and ERBB signaling could reverse the endocrine resistance caused by SMAD4 deletion, but the inhibition rate of the rescue group was weaker compared to the inhibition rate of OHT in MCF7 parental cells (Fig. 7A). We found that OHT could inhibit mTOR signaling (Fig. 5B), which has been reported to promote the autophagic process [29]. Therefore, we investigated whether autophagy is aberrantly activated upon combined inhibition of ER and ERBB activity. ORA using the MSigDB dataset revealed enriched autophagy-associated gene sets in DEGs (Fig. 7B). GSEA using GO gene sets was further performed using RNA-seq data obtained from SMAD4-depleted MCF7 cells treated with LAPA plus OHT. Activation of the autophagy pathway was observed (Fig. 7C). Autophagy-associated markers and pathways were assessed through immunoblot assays. The findings revealed that the combined treatment of LAPA plus OHT significantly induced autophagic processes (Fig. 7D–F and original data). Additionally, validation of autophagy emergence was confirmed by the autophagy flux reporter (Fig. 7G). Next, we investigated whether autophagy inhibition with CQ could enhance the rescue effect of OHT and LAPA in SMAD4-depleted MCF7 cells. According to the results, autophagy inhibition further enhanced the rescue effect of LAPA on endocrine therapy (Fig. 7H, I). To determine whether autophagy inhibition could affect the combined treatment effect of OHT and LAPA, synergistic experiments were performed to determine whether autophagy inhibition could affect the combined treatment effect of OHT and LAPA in SMAD4-depleted MCF7 cells. In the LAPA and OHT plus LAPA groups, cell viability did not appear to be significantly different (Fig. 7J), whereas there was a significant difference when the autophagic process was blocked by CQ (Fig. 7K). The results indicated that autophagy inhibition enhanced the combined treatment effect of LAPA and OHT (Fig. 7L). According to the calculated combination indices, OHT and LAPA exerted synergistic effects when the autophagic process was inhibited (Fig. 7M). These results suggest that the combined inhibition of ER and ERBB signaling leads to increased autophagic dependence of SMAD4-depleted endocrine-resistant cells.

### Combined inhibition of ER, ERBB, and autophagy produced a synergistic effect

**Fig. 8:**
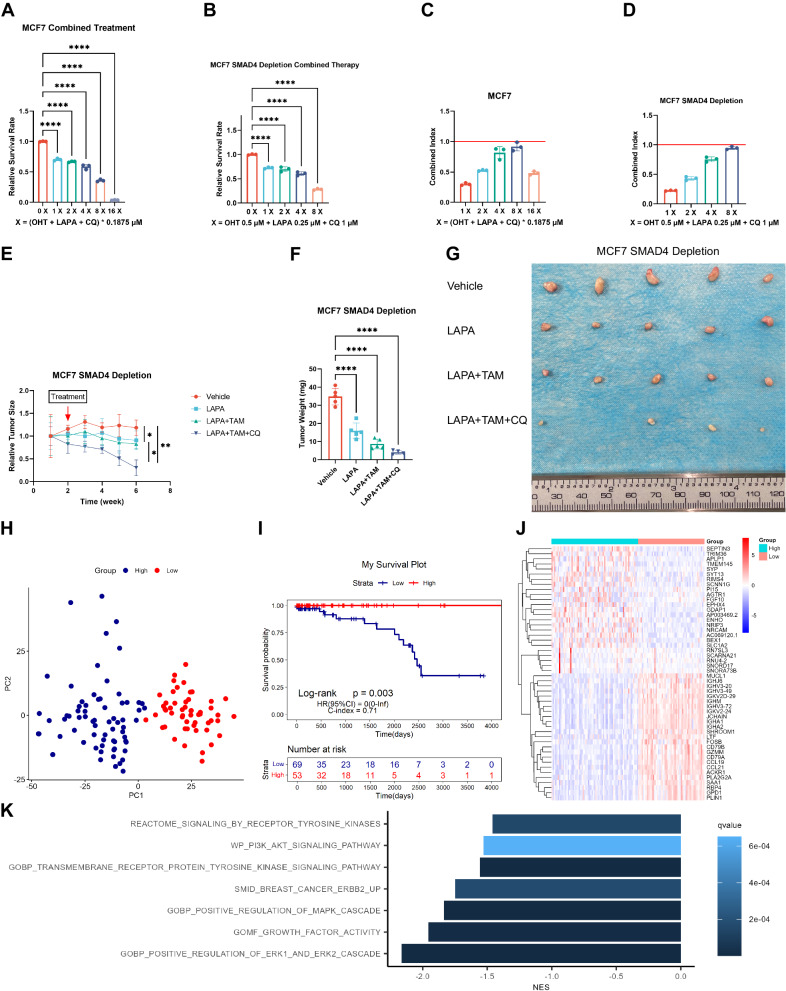
Combined inhibition of ER, ERBB, and autophagy produced a synergistic effect. **A**–**D** Synergy experiments with OHT, LAPA, and CQ in SMAD4 knockout and parental MCF7 cells. A-B, Cell viability of cells treated with OHT in combination with LAPA and CQ. Error bars represent the mean ± SEM of *n* = 3 biological replicates. Statistical significance indicated by asterisks (*p < 0.05, **p < 0.01, ***p < 0.001, ****p < 0.0001, two-way ANOVA). **C**, **D** The bar graph shows the combination indices. Error bars represent the mean ± SEM of *n* = 3 biological replicates. **E** Line graph showing relative tumor size over time in vehicle, LAPA, LAPA + TAM, or LAPA + TAM + CQ treatment. Error bars represent mean ± SEM of *n* = 5 biological replicates. *p*-value (Mann–Whitney *U* test) as indicated. **F** Bar graphs show tumor weight at the end of the experiment. Error bars represent mean ± SEM of *n* = 5 biological replicates. Statistical significance denoted by asterisks (*p < 0.05, **p < 0.01, ***p < 0.001, ****p < 0.0001, Student’s *t* test). **G** Photos show tumor specimens excised at the end of the experiment. **H** The dot plot showing HR + HER2− breast cancer samples being classified into two clusters according to the primary component analysis result. **I** Survival analysis: Low SMAD4 expression correlates with a poor outcome. **J** Heatmap of the top 50 differential gene expressions between high and low SMAD4 expression groups. **K** The bar graph of GSEA shows enriched MSigDB gene sets. The width of the bars is scaled by the normalized enrichment score (NES). The colors of the bars are scaled by the q-value.

We tested the combination of OHT, LAPA, and CQ to determine its efficacy. While each inhibitor alone did not significantly inhibit cell viability at lower concentrations in parental and SMAD4-depleted MCF7 cells (Supplementary Fig. 5C–H), their combined application at lower concentrations resulted in a significant reduction in cell viability (Fig. 8A, B). Additionally, calculated combination indices indicated a synergistic effect (Fig. 8C, D). Then, we validated these results in vivo, and the results showed that the three-drug combination panel significantly inhibited tumor growth (Fig. 8E–G). Finally, we investigated the clinical significance of SMAD4 using RNA-seq data from TCGA HR + HER2− patient samples. Initially, a principal component analysis was conducted to stratify patients into high and low SMAD4 expression groups (Fig. 8H). Subsequently, survival analysis revealed a significant survival advantage in the SMAD4 high expression group (Fig. 8I). Expression difference analysis between the high and low SMAD4 expression groups was then performed (Fig. 8J). GSEA using MSigDB gene sets unveiled inhibited ERBB signaling (Fig. 8K), suggesting a subgroup of patients with low SMAD4 expression exhibiting elevated ERBB activity. Hence, the combination of OHT, LAPA, and CQ may represent an effective treatment option for patients with SMAD4 depletion.

## Discussion

Several mechanisms underlying the resistance of HR + HER2− breast cancer to endocrine therapy have been identified in recent decades. These include abnormal ER, ERBB, and mTOR signaling. Treatment of endocrine resistance in cancer has been attempted in a variety of ways. Currently, only two types of agents are available for this purpose, mTOR inhibitors and CDK4/6 inhibitors, and further therapeutic development is required [30–33]. The development of endocrine resistance is the result of multiple factors, making it impossible to overcome endocrine resistance with a single-factor approach. Therefore, it will be helpful to gain a deeper understanding of the dynamic regulatory process involving multiple mechanisms to solve the problem of endocrine resistance.

Genome-wide CRISPR screening is a powerful genetic screening tool that reveals abundant information about gene perturbations for specific phenotypes [34]. Using genome-wide CRISPR screening in MCF7 cells, ARID1A was shown to play a critical role in endocrine resistance [19, 35]. Here, we used this method in T47D cells to further characterize gene alterations associated with endocrine resistance, and we found that SMAD4 deficiency confers resistance to OHT. Furthermore, we observed that hormone therapy reduced SMAD4 expression and that SMAD4 deficiency predicted a poor clinical outcome of endocrine therapy. SMAD4 has been reported to function as a transcriptional corepressor of the ER [17]. We performed RNA-seq to investigate the underlying mechanism and confirmed that the depletion of SMAD4 led to abnormal ER activity. However, the activated ER signaling pathway in SMAD4-depleted MCF7 cells could be suppressed by OHT, suggesting that other mechanisms are involved in SMAD4 depletion-induced endocrine resistance. Our transcriptomic profile also confirmed the role of the mTOR pathway in abnormal ER activity, as previously described [26]. mTOR is a known downstream signaling pathway of the ERBB pathway [36], and previous studies suggested that ERBB signaling may play a critical role in mediating endocrine therapy resistance by modulating ER activity [37–39], which prompted us to investigate the relationship between SMAD4 and ERBB signaling. In this study, we found that EGFR/HER2 was upregulated in SMAD4-depleted cells. Our study indicates that SMAD4 is a critical factor involved in the coordination of ER and ERBB functions. Previous studies have shown that crosstalk between the ER and ERBB pathways functionally contributes to acquired endocrine-resistant breast cancer [38, 40]. However, the combined inhibition of ER and ERBB signaling by OHT and LAPA did not completely reverse endocrine resistance in our study. Combined OHT and LAPA treatment significantly inhibited mTOR, which increased autophagy dependence. Therefore, autophagy inhibition significantly enhanced the combined treatment effect of OHT and LAPA. The combination of OHT, LAPA, and CQ has a synergistic effect, suggesting that the co-inhibition of ER and ERBB signaling, and autophagy may be a better treatment option for patients with endocrine resistance.

While EGFR and HER2 are members of the ERBB family, which also includes HER3 and HER4 [41], our study solely focused on evaluating EGFR and HER2. Future research should aim to clarify the roles of HER3 and HER4 to provide a comprehensive understanding of ERBB signaling. ER and ERBB signaling controls a variety of pathways and functions. Only mTOR was used in our study to understand endocrine resistance. Further research is needed to determine the function of other downstream mechanisms. In addition to the weakness mentioned above, this study did not illustrate the exact mechanism on how endocrine therapy represses SMAD4 expression and how SMAD4 regulates EGFR/HER2 expression, these works will be completed in the upcoming future. While previous studies have reported the role of ER, and ERBB pathways in endocrine resistance, our study clarifies the role of SMAD4 in orchestrating ER, and ERBB signaling pathways, which provides a novel perspective on the development of acquired endocrine resistance and suggests that combined inhibition of ER activity, ERBB signaling, and autophagic process may be a rational therapeutic strategy for patients with acquired endocrine-resistant breast cancer. Ultimately, our goal is to promote the use of SMAD4 as a predictive, diagnostic, and therapeutic molecular marker to aid in precision medicine for breast cancer.

## Materials and methods

### Cell lines and agents

The HR + HER2− luminal breast cancer cell lines MCF7 (ATCC, Manassas, VA, USA) and T47D (ATCC, Manassas, VA, USA) were cultured in Dulbecco’s modified Eagle’s medium (DMEM, Gibco, USA) supplemented with 10% fetal bovine serum (FBS, ExCell, China), 50 U/mL penicillin, and 50 ng/mL streptomycin. Lenti-X 293 T cells (Clontech, Japan) were cultured in DMEM supplemented with 10% FBS. Cells were cultured in a humidified atmosphere with normal oxygen levels (5% CO_2_, 37 °C). All cell lines were verified by short tandem repeat profiling before use and were routinely tested for mycoplasma contamination. The agents used in this study are listed in Supplementary Table 5.

### Genome-wide CRISPR screening

The human Brunello CRISPR knockout pooled library was donated by David Root and John Doench (Addgene, 73179, USA) and contains 76,441 gRNAs targeting 19,114 genes in the human genome [42]. For screening, a Brunello library with the lentiCRISPR v2 backbone was selected for packaging into a lentiviral library. Viral titers were measured using the Lenti-X qRT-PCR Titration Kit (Clonetech, Japan). T47D breast cancer cells were infected at a multiplicity of infection (MOI) = 0.3, resulting in only one gRNA being integrated into the genome per cell. After 24 h of infection, the infected cells were subjected to antibiotic selection by exposure to 1 μg/mL puromycin for 7 days to obtain a mutant cell pool. Mutant cells were cultured for several days to obtain sufficient cells for this screen. A total of 4×10^7^ mutant cells were collected as a baseline group prior to drug selection. The same number of mutant cells were then treated with vehicle (DMSO group) or 1 μM OHT (OHT group) for 21 days. A total of 4×10^7^ cells per experimental group were collected after treatment, and genomic DNA was then extracted from all three groups. Approximately 270 μg of genomic DNA per group, corresponding to 4×10^7^ T47D cells, was amplified (5 μg per reaction, 54 times) with specific primers using NEBNext Ultra II Q5 Hot Start High-Fidelity 2X Master Mix (NEB, USA). Primers are listed in Supplementary Table 5. PCR was performed for 25 cycles according to the manufacturer’s instructions. The presence of 162 base pair (bp) PCR products was verified by agarose gel electrophoresis. All the verified PCR products were then pooled and purified using the TIANgel Maxi purification kit (TIANGEN, China). The purified PCR products were then subsequently sequenced on an Illumina platform (Novogene Technology Co., Ltd., China). Raw reads were depleted of low-quality sequences using fastp [43], and reads were counted using MAGeCK [44, 45], followed by gene ranking.

### RNA-seq

To investigate transcriptomic changes, we performed two rounds of RNA-seq. In the first round, parental or SMAD4 knockout MCF7 cells were treated with DMSO or 1 μM OHT for 24 h were used. In the second round, parental MCF7 cells or SMAD4 knockout MCF7 cells treated with DMSO or 1 μM LAPA + 1 μM OHT for 24 h were used. Three independent experimental replicates were performed. Total RNA was extracted with TRIzol (Invitrogen, USA) according to the manufacturer’s instructions. RNA integrity and concentration were measured using the RNA Nano 6000 Assay Kit (Agilent Technologies, USA) on a Bioanalyzer 2100 system. Sequencing libraries were prepared from 2 μg of RNA per sample using the NEBNext® Ultra II RNA Library Prep Kit for Illumina® (NEB, USA). Sequencing was performed on an Illumina platform (Novogene Technology Co., Ltd., China). Raw sequenced reads were trimmed using fastp [43] and then mapped to the human reference genome (GRCh38 from the Ensembl genome browser) using Rsubread [46]. Read counts for the genes in each sample were determined using featureCount from Rsubread, and differential expression analysis was performed using DeSeq2 [47]. Genes with an adjusted *p*-value < 0.05 and |log2FC| > 0.5 were identified as DEGs. The corresponding heatmap was generated using the R package pheatmap. ORA and (GSEA) were performed with ClusterProfiler [48] using gene sets from GO, KEGG, and MsigDB.

### Vector construction and lentivirus transduction

To construct the lentiviral cDNA overexpression vector, human SMAD4 cDNA (NCBI, NM_005359) was amplified by PCR and cloned into the LV-ECMV-PURO vector, modified from pCDH-CMV-MCS-EF1a-CopGFP-T2A-Puro (System Biosciences, USA). For the lentiviral shRNA knockdown vector, Invitrogen BLOCK-iT RNAi Designer [49] was used to design the shRNA sequence of SMAD4. A non-targeting shRNA was used as a negative control. The lentiviral shSMAD4 vector was generated by ligation of hybridized oligos into the LV-ECMV-RFP-shRNA-PURO vector (modified from LV-ECMV-PURO) using T4 DNA ligase (Sangon Biotech, China). To generate the lentiviral CRISPR knockout vector, single sgRNAs targeting the SMAD4 gene were designed using CRISPick [50]. A non-targeting sgRNA was used as a control. The lentiviral SMAD4 knockout vector was generated by ligation of hybridized oligos into the lentiCRISPRv2 puro (Addgene, 98290, USA) as previously described [51]. For lentivirus production, Lenti-X 293 T cells were seeded in 10 cm dishes 24 h prior to transfection. A mixture of 2 μg pMD2.G (Addgene, 12259, USA) envelope vector, 4 μg pspAX2 (Addgene, 12260, USA) packaging vector, and 5 μg lentiviral vector with 1 mL optiMEM (Gibco, USA) and 20 μL Lipofectamine 3000 (Thermo Fisher, USA) transfection reagent was then added to the cells and incubated for 20 min. The medium was refreshed 6 h after transfection, and the supernatant of 293T cells containing lentivirus was collected 48 h after transfection. For stable overexpression and knockdown cell line construction, viral supernatant supplemented with 8 μg/mL polybrene (Beyotime, China) was used to transduce MCF7 and T47D cells, followed by selection with 1 μg/mL puromycin for 7 days (Beyotime, China). To generate independent non-sister SMAD4 knockout clonal cell lines, MCF7 and T47D cells were infected with the indicated lentivirus-containing polybrene for 48 h. Positively transduced cells were obtained by selection with 1 μg/mL puromycin for 7 days, and single cells were plated in two 96-well plates for each sgRNA at 7 days post-infection by serial dilution. The SMAD4-depleted clones were validated by immunoblotting. The DNA sequences used in this study are listed in Supplementary Table 5.

### RNA extraction and real-time quantitative PCR (RT-qPCR)

RNA was isolated using the FastPure Cell/Tissue Total RNA Isolation Kit V2 (Vazyme, China), and its concentration and quality were assessed using a NanoDrop 2000 spectrophotometer (Thermo Fisher Scientific, USA) before cDNA synthesis using RT Master Mix for qPCR II (MCE, China) with a maximum of 1 μg total RNA per sample. RT-qPCR was performed on the CFX96 system (Bio-Rad, USA) using SYBR Green qPCR Master Mix (MCE, China) and sets of gene-specific primers. Gene expression levels were evaluated by the ∆∆CT method (CT, threshold cycle) and normalized to the level of GAPDH in each sample. The primers used in this study are listed in Supplementary Table 5.

### Immunoblotting

Total protein was extracted from cultured cells using cell lysis buffer (Beyotime, China), quantified using a BCA protein assay kit (Beyotime, China), subjected to electrophoresis on SDS-polyacrylamide gels (20 μg per sample), and transferred to polyvinyldifluoride (PVDF) membranes (Bio-Rad, USA). The membranes were blocked with 3% bovine serum albumin or 5% skim milk in Tris-buffered saline and Tween 20 (TBST) for 60 min at room temperature and then incubated with primary antibodies (dilutions ranging from 1:1000 to 1:5000) at 4 °C overnight. The membranes were then washed with TBST three times and incubated with the indicated secondary antibodies for 1 h at room temperature. Chemiluminescence signals were generated using the BeyoECL Star Kit (Beyotime, China) and visualized using the ChemiDoc MP Imaging System (Bio-Rad, USA). Before exposure, the membranes were cut to show only the gel containing the bands of interest. The immunoblot images in the figure have been cropped for presentation. Fiji was used to quantify the intensity of the bands [52]. The same standard rectangle was used for each sample and for background correction for each lane. The antibodies used in this study are listed in Supplementary Table 5.

### Cell viability assay

A CCK8 assay (MCE, China) was used to assess cell viability according to the manufacturer’s instructions. Cells were seeded in 96-well plates at a density of 8000 cells/well and cultured overnight before drug addition. For dose-dependent effect studies, cells were treated with different concentrations of OHT (0, 1, 2, 4 μM) or FVE (0, 0.1, 0.2, 0.4, 0.8 μM). For mTOR signaling pathway rescue assays, cells were treated with various concentrations of OHT or FVE as well as 1 μM EVE. For ERBB signaling pathway rescue assays, cells were treated with different concentrations of OHT or FVE plus 1 μM LAPA. For synergy experiments with OHT and LAPA, cells were treated with OHT (0, 1, 2, 4, 8 μM), LAPA (0, 1, 2, 4, 8 μM), or OHT plus LAPA (0 X, 1 X, 2 X, 4 X, 8 X, where X (1 μM OHT + 1 μM LAPA) was defined as the concentration of the drug combination) either alone or in combination with a constant amount of CQ (1 μM). For synergistic experiments with OHT, LAPA and CQ, MCF7 parental cells were treated separately with OHT (0, 0.5625, 1.125, 2.25, 4.5, 9 μM), LAPA (0, 0.5625, 1.125, 2.25, 4.5, 9 μM), CQ (0, 0.5625, 1.125, 2.25, 4.5, 9 μM), or OHT plus LAPA and CQ (0 X, 1 X, 2 X, 4 X, 8 X, where X (0.1825 μM OHT + 0.1825 μM LAPA + 0.1825 μM CQ) was defined as the concentration of the drug combination). MCF7 SMAD4 knockout cells were treated separately with OHT (0, 1.5, 3, 6, 12 μM), LAPA (0, 0.75, 1.5, 3, 6 μM), CQ (0, 3, 6, 12, 24 μM), or OHT plus LAPA and CQ (0 X, 1 X, 2 X, 4 X, 8 X, where X (0.5 μM OHT + 0.25 μM LAPA + 1 μM CQ) was defined as the concentration of the drug combination).Cells were incubated at 37 °C for 72 h before cell viability was quantified. The culture medium was replaced with DMEM containing 10% CCK8 solution and incubated at 37 °C for 2 h. The OD values were quantified at 450 nm using an enzyme-labeling instrument (Thermo Fisher Scientific, USA). Cell viability (%) was normalized to control viable cells to calculate the relative survival rate. The Chou-Talalay method [53] was used to calculate combination indices using CompuSyn software. According to the Chou-Talalay combination index theorem, additive effects of the drug combinations were defined as CI = 1, synergistic effects as CI < 1, and antagonistic effects as CI > 1. Experiments were performed in triplicate, and the average of the control group was set at 100%.

### Colony formation assay

Colony formation assays were performed by plating 500–2000 cells plated on 24-well plates. Media containing the indicated concentrations of OHT were replaced every 3 days, and cells were allowed to grow for 10–14 days. Paraformaldehyde was used to fix the cells, 0.2% crystal violet solution was used for staining, and photographs were taken.

### Confocal microscopy

SMAD4-depleted MCF7 cells were plated onto glass-bottom culture dishes and infected with mRFP-GFP-LC3 lentivirus for 48 h. Subsequently, cells were treated with either DMSO or OHT + LAPA for 24 h, fixed in 4% paraformaldehyde, and stained with DAPI for nuclear visualization. Finally, the autophagic flux was visualized and monitored using confocal laser scanning microscopy (STELLARIS 8, Leica, USA).

### Animal experiments

All animal experiments were approved by the Ethics Committee of the First Affiliated Hospital of Chongqing Medical University. Five-week-old female athymic BALB/c nude mice, purchased from Vital River Laboratories (Beijing), were maintained until 6 weeks of age in the specific pathogen-free (SPF) animal facility of the Laboratory Animal Resource Center of Chongqing Medical University. One week before injection, 30 μg of estradiol (cypionate) dissolved in 30 μL of corn oil (Beyotime, China) was injected intramuscularly to allow the estrogen-dependent MCF7 cells to proliferate. Estradiol supplementation was performed every 7 days during the study. A total of five million SMAD4-KO or parental MCF7 cells were resuspended in a 1:1 solution of PBS and BD Matrigel®, High Concentration (BD Bioscience, USA), and injected orthotopically into the fourth pair of mammary fat pads of nude mice. For the phenotype experiment, tumor-bearing mice were randomized (using the RAND function in Microsoft Excel) into the TAM and FVE groups as indicated (*n* = 5/group). The statistician was blinded to the treatment group allocation. After 1 week, TAM or FVE was administered subcutaneously in a corn oil solution at a dose of 250 mg/kg per week for 6 weeks. For the combined therapy experiment, tumor-bearing mice were randomized (using the RAND function in Microsoft Excel) into the Vehicle, LAPA, LAPA + TAM, and LAPA + TAM + CQ groups as indicated (*n* = 5/group). The statistician was blinded to treatment group allocation. After 1 week, LAPA, TAM or CQ were administered separately or in combination subcutaneously in a corn oil solution at a dose of 250 mg/kg per week for 6 weeks. Tumor volume (mm^3^) was monitored weekly using the formula width^2^ × length/2. The relative tumor size of each tumor was defined as the ratio of the volume at a given time to the volume before treatment. Mice were euthanized by cervical dislocation after an overdose of anesthesia at the experimental endpoint. The xenografts were then excised and weighed. IHC staining was performed with rabbit anti-SMAD4 polyclonal antibody at a dilution of 1:150, as previously described [22]. Representative photographs were taken with a Leica microscope (Leica, Germany).

### Bioinformatic analysis of publicly available data

RNA-seq data from breast cancer patients from The Cancer Genome Atlas (TCGA) were obtained from the Genomic Data Commons data portal website [24]. RPPA data from CCLE, and Gene dependency data from PRISM were obtained from the depmap portal [54]. Expression analysis, Correlation analysis, and survival analysis were performed using R. The association of gene expression with clinical prognosis was analyzed using K-M Plotter [25].

### Statistics

Statistical analyses performed in this study were performed using GraphPad Prism 9.0, R (v. 4.1.3), and RStudio (v. 2022.07.2 + 576). Figures and legends show exact numbers (N), number of independent experiments with similar results, statistical tests, and *p*-values. Error bars in figures indicate S.D. or S.E.M. Two-tailed Student’s *t* test was used for one-factor, one-level variables. Two-way ANOVA with Dunnett’s or Sidak’s multiple comparison test was used for one-factor, multiple-level variables. Two-way ANOVA with Dunnett’s or Sidak’s multiple comparison test was used for data with two-factor variable data. A *p*-value < 0.05 was considered statistically significant.

### Supplementary information

Supplementary figures

Supplementary file 1

Supplementary Table 1

Supplementary Table 2

Supplementary Table 3

Supplementary Table 4

Supplementary Table 5

original data

## Data Availability

The high-throughput sequencing data generated in this study were deposited in the National Center for Biotechnology Information Sequence Read Archive (SRA) database with BioProject accession numbers PRJNA911670 and PRJNA911854. The data set analyzed in the current study was obtained from the Gene Expression Omnibus (GSE123283). Additional data are available in the article or in the Supplementary Information. The original data are available with this article. The experimental material is available upon reasonable request from the corresponding author.
