# Supplementary material for: SMAD4 depletion contributes to endocrine resistance by integrating ER and ERBB signaling in HR + HER2− breast cancer
Source: Cell Death Dis. 2024 Jun 24;15(6):444. doi: 10.1038/s41419-024-06838-9 (PMC11196642; doi:10.1038/s41419-024-06838-9)
Supplement: Supplementary file 1 — Supplementary figures [file 41419_2024_6838_MOESM1_ESM.pdf]

SUPPLEMENTARY FIGURES

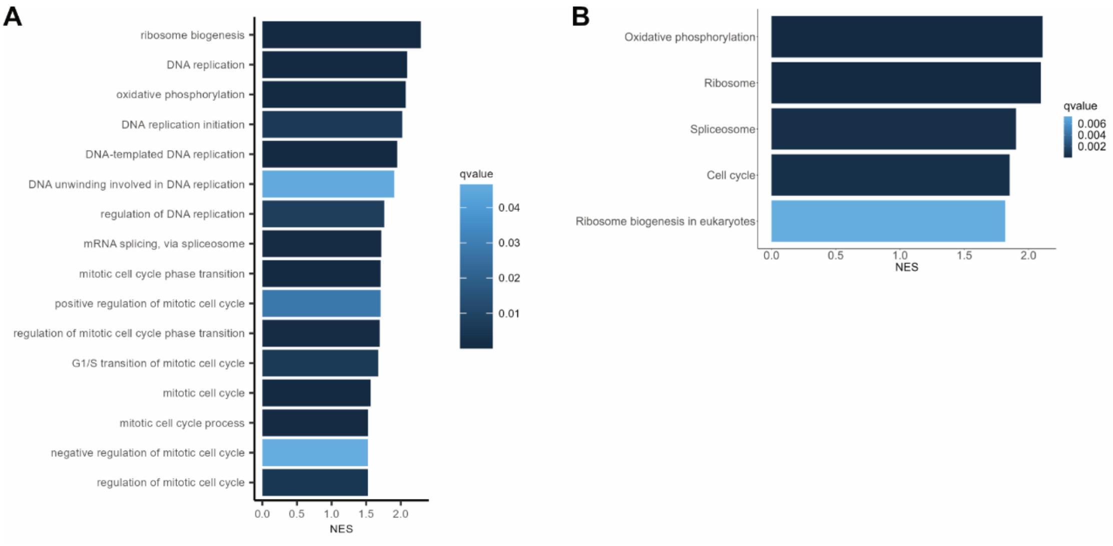

Supplementary Fig. 1 CRISPR screening identifies SMAD4 as a gene involved in endocrine resistance. A The bar graph of GSEA shows enriched GO gene sets. The width of the bars is scaled by the normalized enrichment score (NES). The colors of the bars are scaled by the q-value. **B** The bar graph of GSEA shows enriched KEGG gene sets. The width of the bars is scaled by the normalized enrichment score (NES). The colors of the bars are scaled by the q-value.

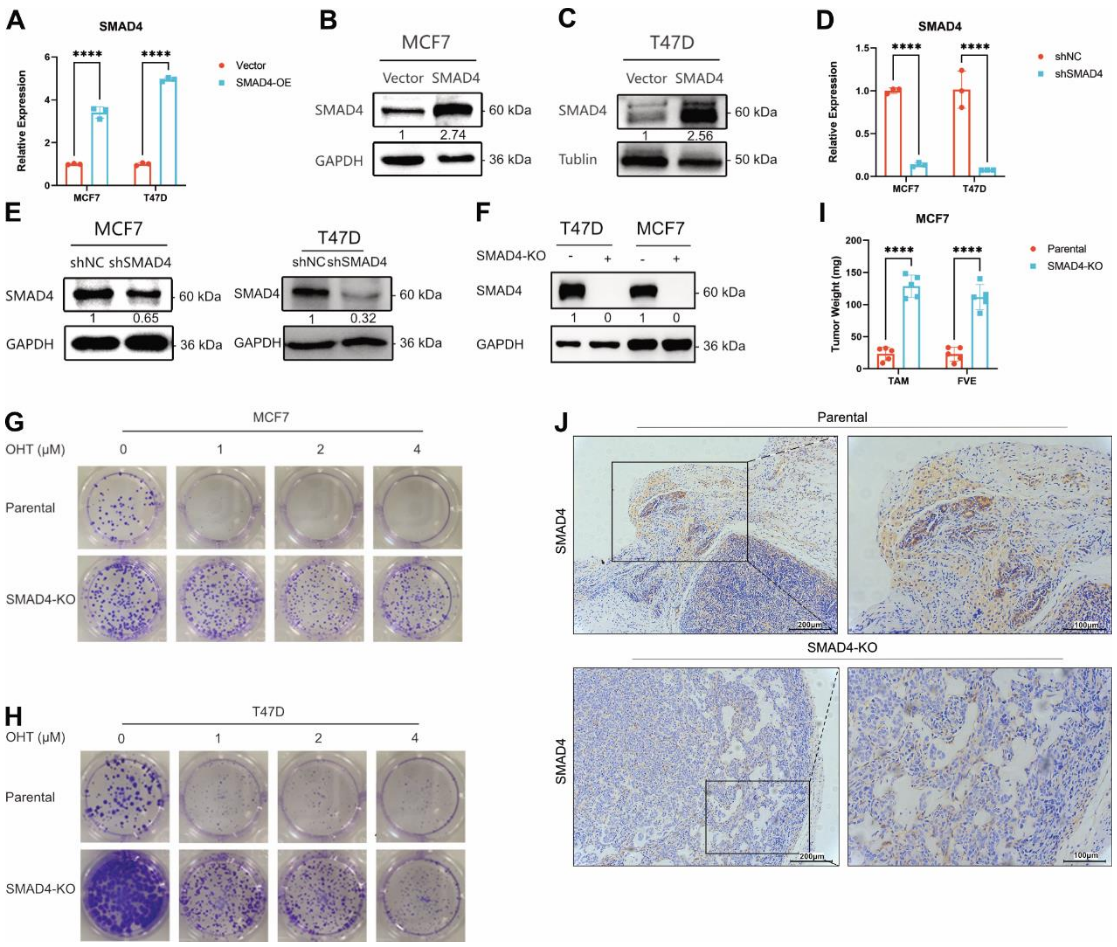

Supplementary Fig. 2 Downregulation of SMAD4 contributes to endocrine resistance. A SMAD4 mRNA expression in SMAD4-overexpressing and control MCF7/T47D cells was determined by RT-qPCR. Error bars represent the mean ± SEM of n = 3 biological replicates. *p < 0.05, **p < 0.01, ***p < 0.001, ****p < 0.0001 (two-way ANOVA). **B-C** SMAD4 protein expression in SMAD4-overexpressing and control MCF7/T47D cells was determined by immunoblot. GAPDH/tubulin was used as a loading control. **D** SMAD4 mRNA expression in SMAD4 knockdown and control MCF7/T47D cells was determined by RT-qPCR. Error bars represent the mean ± SEM of n = 3 biological replicates. *p < 0.05, **p < 0.01, ***p < 0.001, ****p < 0.0001 (two-way ANOVA). **E** SMAD4 protein expression in SMAD4 knockdown and control MCF7 cells was determined by immunoblot. GAPDH was used as a loading control. **F**

SMAD4 protein expression in SMAD4 knockdown and parental T47D cells was determined by immunoblot. GAPDH was used as a loading control. **G-H** Colony formation assay in SMAD4 KO and parental MCF7/T47D cells treated with OHT for 14 days. **I** Bar graphs show tumor weight at the end of the experiment. Error bars represent the mean ± SEM of n = 5 biological replicates. *p < 0.05, **p < 0.01, ***p < 0.001, ****p < 0.0001 (Student's t-test). **J** IHC staining of SMAD4 expression in xenografts (100 X; 200 X). The expression of SMAD4 in xenografts was significantly decreased in the SMAD4 KO group compared to the parental group. Enlarged images on the right and thumbnails on the left. Scale bars, 100 μm; 200 μm.

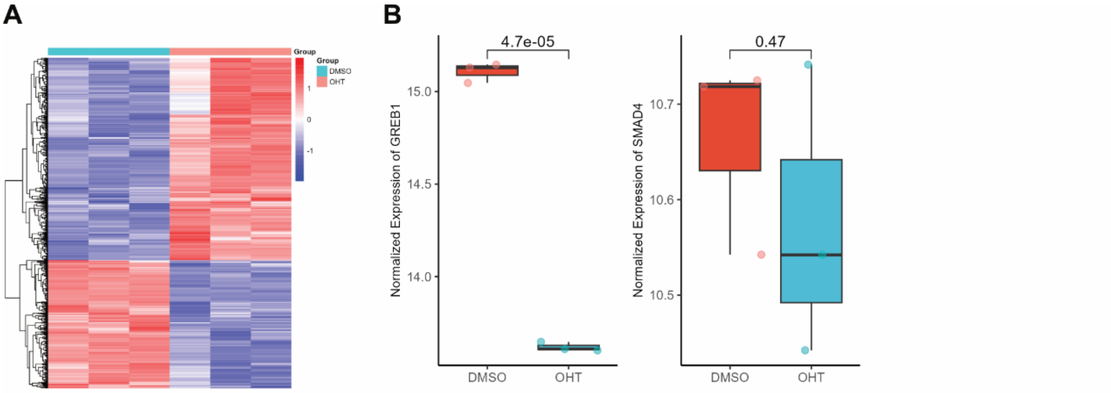

Supplementary Fig. 3 Endocrine therapy-induced SMAD4 downregulation is independent of ER signaling. A Heatmap of differential gene expression in parental vs. SMAD4 knockout (KO) MCF7 cells (absolute log2-fold change > 1, Benjamini-Hochberg adjusted P < 0.05). A total of 417 of these genes showed significant upregulation, while 264 genes showed significant downregulation in the SMAD4 KO group. **B** Bar graph showing normalized expression of GREB1 and SMAD4 in OHT and DMSO treated MCF7 cells.

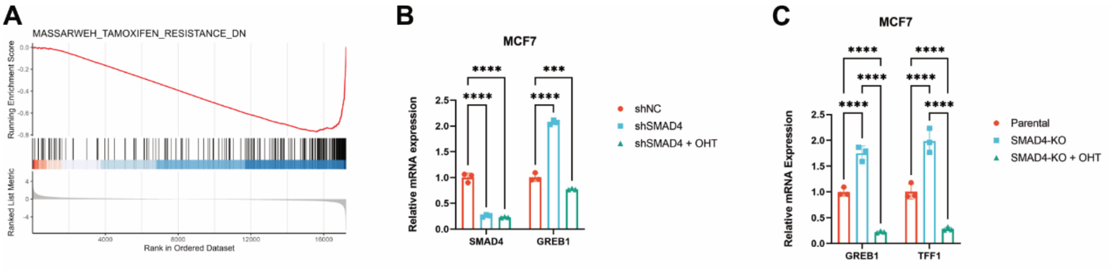

Supplementary Fig. 4 Activation of ER signaling due to SMAD4 depletion is not the sole factor contributing to endocrine resistance. **A** GSEA enrichment plots depict the enriched gene sets in SMAD4 knockout (KO) MCF7 cells treated with OHT compared to those treated with DMSO. A red horizontal bar transitioning to blue indicates the shift from positively correlated genes (red) to negatively correlated genes (blue). **B** SMAD4 and GREB1 mRNA expression levels in MCF7 cells were assessed using RT-qPCR following SMAD4 knockdown (shSMAD4) or SMAD4 knockdown combined with 1 µM OHT treatment for 24 h (shSMAD4 + OHT). Error bars denote the mean ± SEM of n = 3 biological replicates. Statistical significance was determined using two-way ANOVA, with *p<0.05, **p<0.01, ***p<0.001, and ****p<0.0001. **C** GREB1 and TFF1 mRNA expression in MCF7 cells was determined by RT-qPCR following SMAD4 knockout (SMAD4-KO) or SMAD4 knockout combined with 1 µM OHT treatment for 24 h (SMAD4-KO + OHT). Error bars represent the mean ± SEM of n = 3 biological replicates. Statistical significance was assessed using two-way ANOVA, with *p<0.05, **p<0.01, ***p<0.001, and ****p<0.0001.

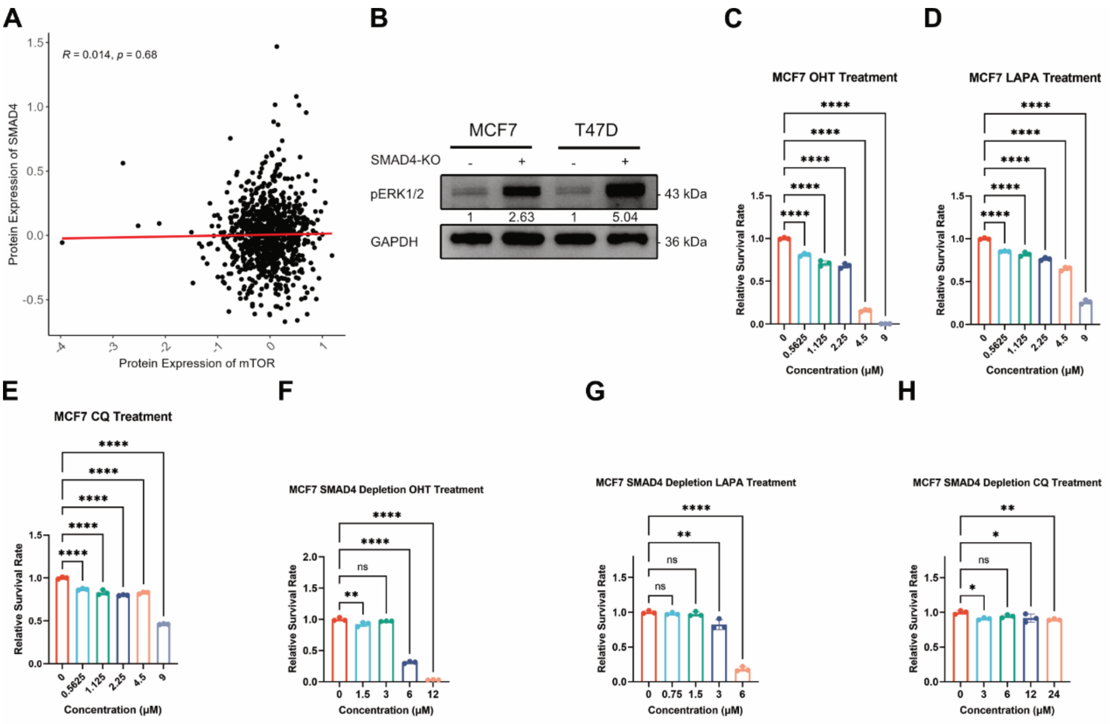

Supplementary Fig. 5 A The dot plot illustrates the correlation in protein expression between SMAD4 and mTOR, with correlation coefficient and p-value indicated. **B** Immunoblot analysis was conducted to assess the protein expression of pERK1/2 in SMAD4 knockout and parental MCF7/T47D cells, with GAPDH serving as the loading control. **C-H** Synergy experiments involving OHT, LAPA, and CQ were performed in both SMAD4 knockout and parental MCF7 cells. Cell viability of parental and SMAD4-depleted MCF7 cells following treatment with OHT (C, F), LAPA (D, G), or CQ (E, H) was evaluated. Error bars denote the mean ± SEM of n = 3 biological replicates. Statistical significance was determined using two-way ANOVA, with $*p<0.05$, $**p<0.01$, $***p<0.001$, and $****p<0.0001$.
