# Supplementary material for: SMAD4 depletion contributes to endocrine resistance by integrating ER and ERBB signaling in HR + HER2− breast cancer
Source: Cell Death Dis. 2024 Jun 24;15(6):444. doi: 10.1038/s41419-024-06838-9 (PMC11196642; doi:10.1038/s41419-024-06838-9)
Supplement: Supplementary file 2 — Supplementary file 1 [file 41419_2024_6838_MOESM2_ESM.docx]

**Supplementary file 1. Expression and survival difference analysis for candidate genes.**

CCNC:


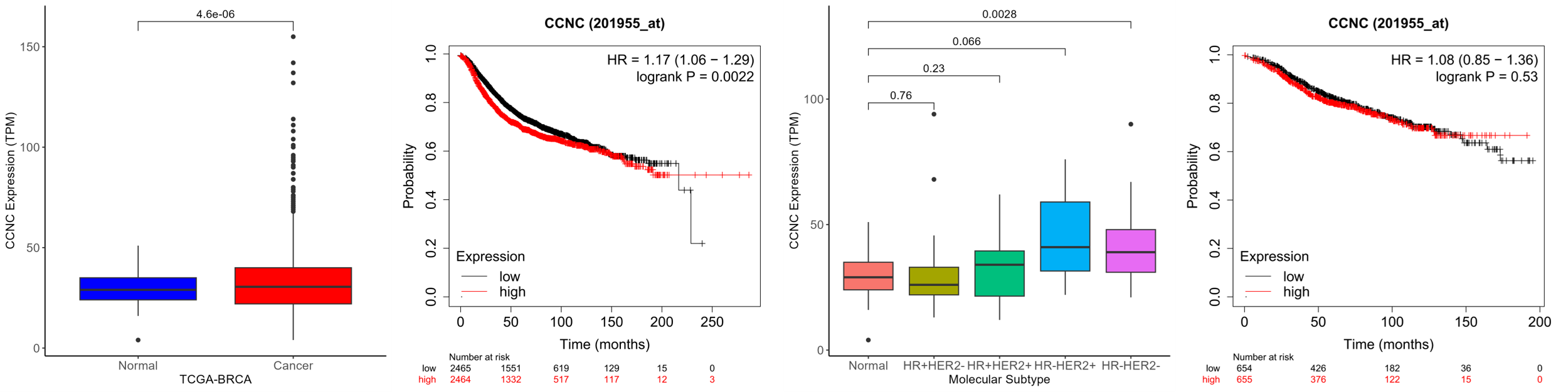


FPGS:


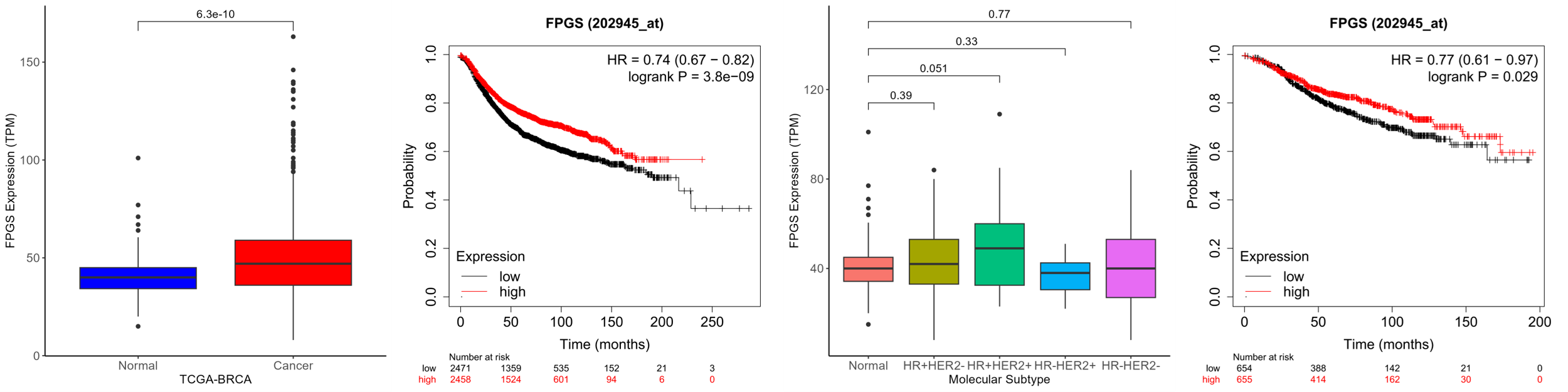


MED12:


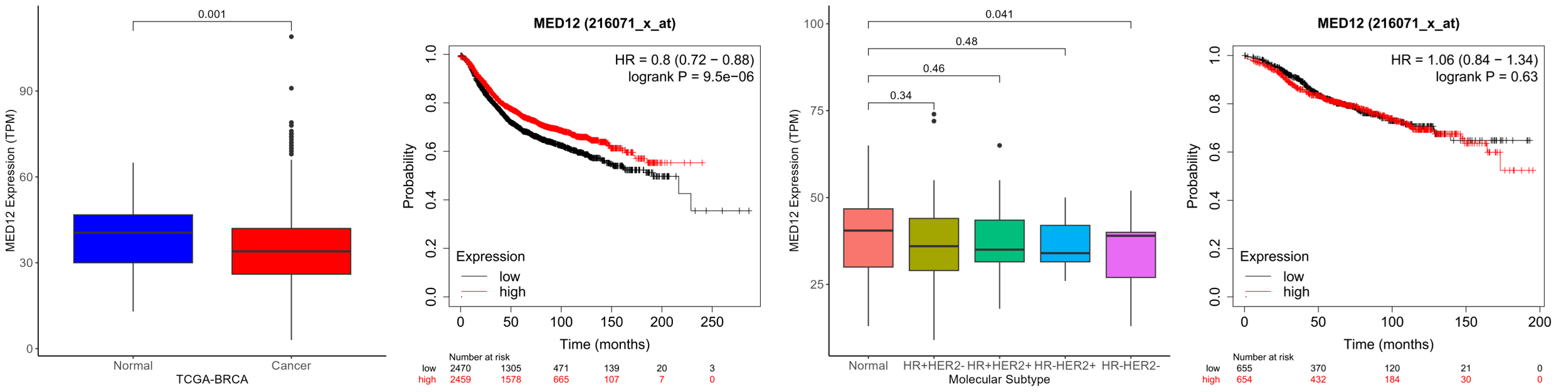


NDUFA6:


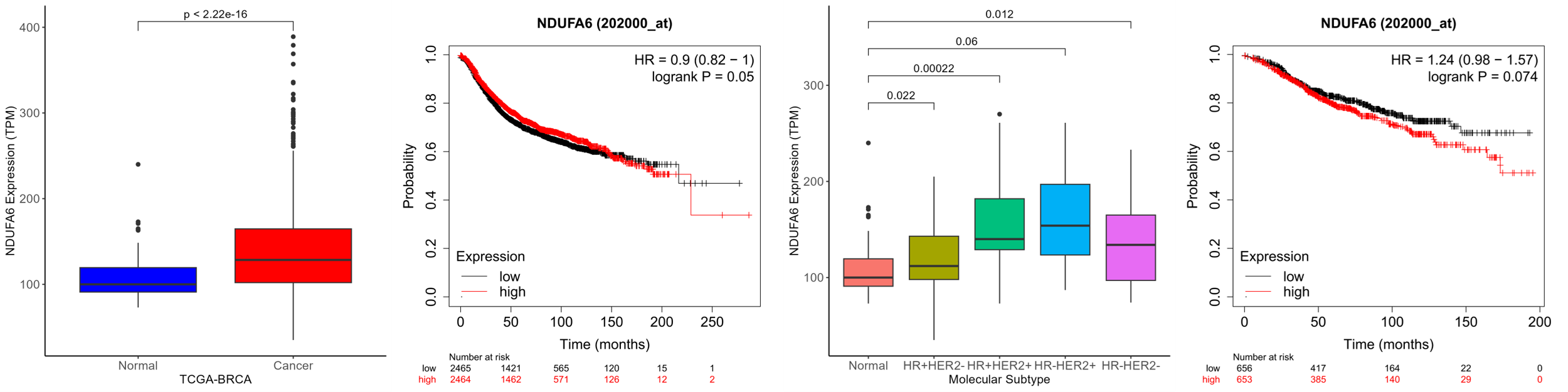


STRAP:


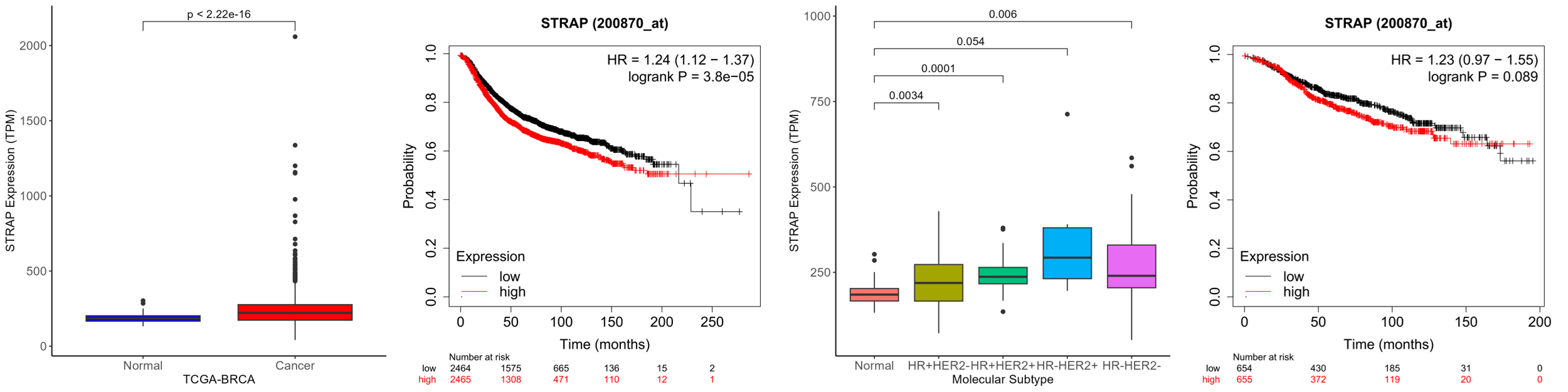


TAF6L:


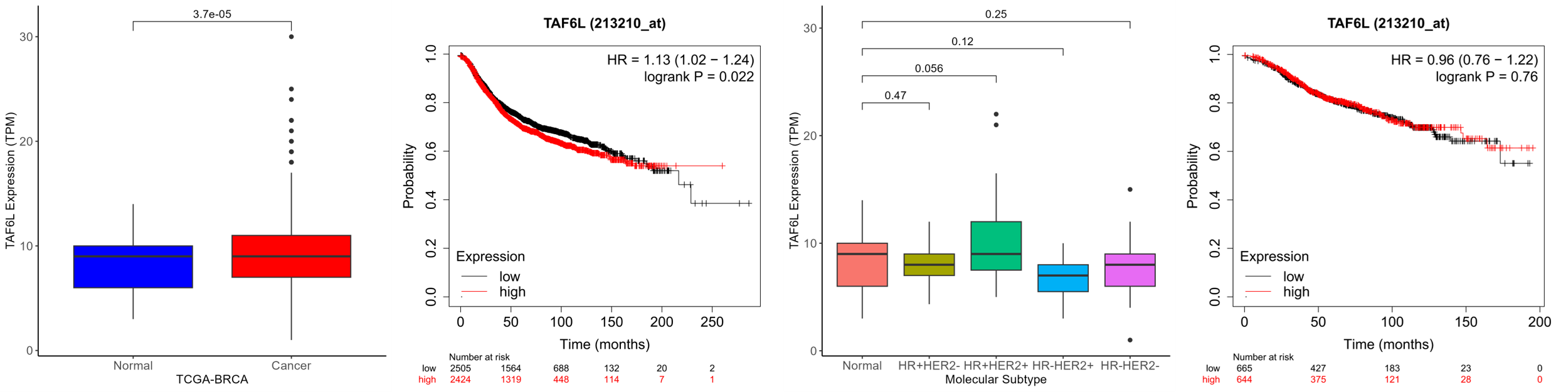


TAF10:


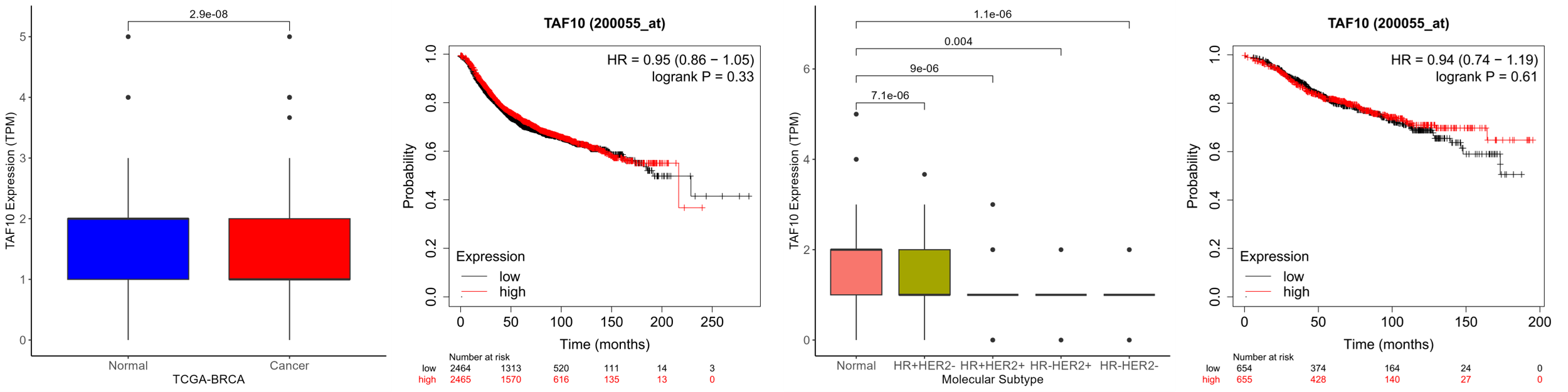


TIMMDC1:


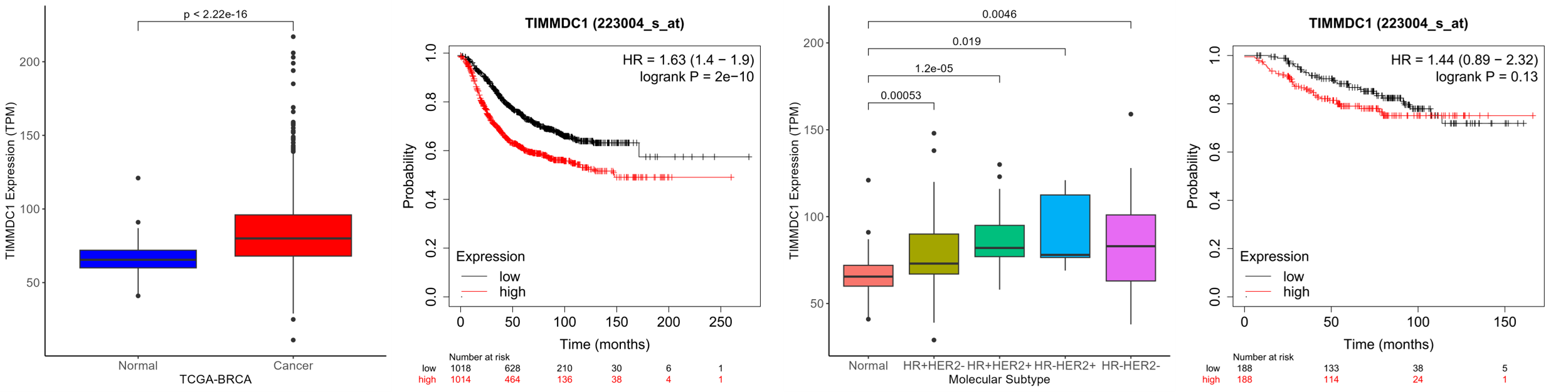


Box plots show the expression differences between normal (n = 102) and cancerous (n = 1022) tissues, as well as among normal (n = 102), HR+HER2- (n = 486), HR+HER2+ (n = 269), HR-HER2+ (n = 76), and HR-HER2- (n = 191) cancer tissues in the TCGA-BRCA dataset. Expression data were normalized to transcripts per kilobase million (TPM) values. p-values (Student's t-test) are indicated. The plots include the median line, box borders (25th and 75th percentiles), and whiskers (1.5 times the interquartile range). Survival analysis using the K-M Plotter was conducted to evaluate the correlation between gene expression and survival in breast cancer patients, specifically those with HR+ breast cancer treated with endocrine therapy.
