# Supplementary material for: SMAD4 depletion contributes to endocrine resistance by integrating ER and ERBB signaling in HR + HER2− breast cancer
Source: Cell Death Dis. 2024 Jun 24;15(6):444. doi: 10.1038/s41419-024-06838-9 (PMC11196642; doi:10.1038/s41419-024-06838-9)

**Original data. Original data of western blot**

Supplementary Fig. 2

B:


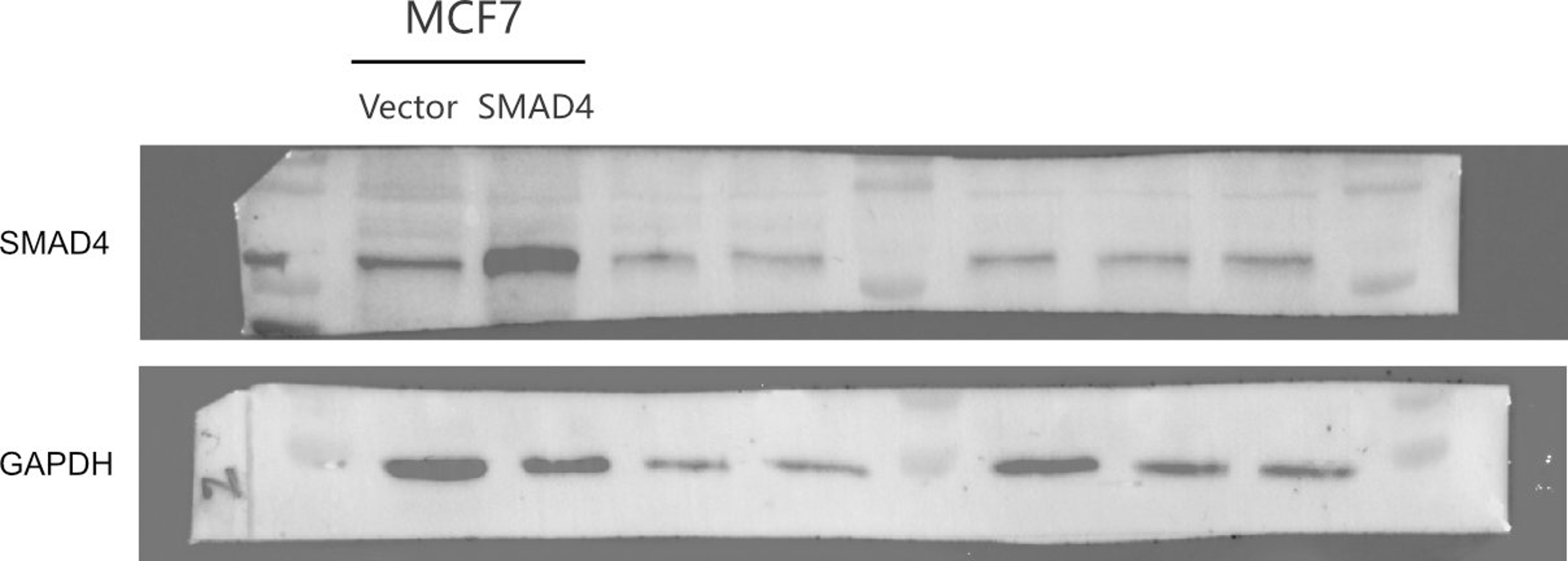


C:


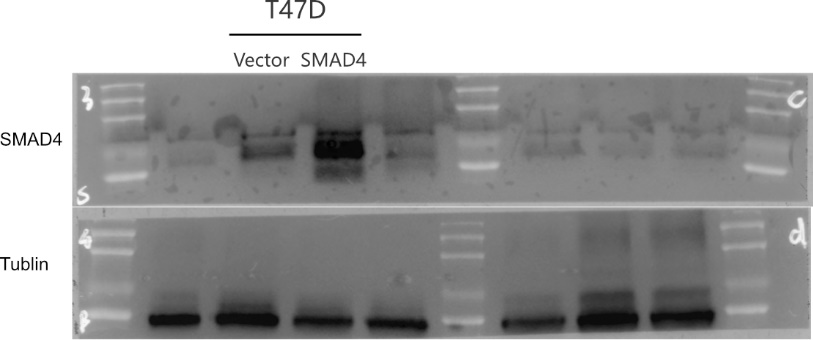


E:


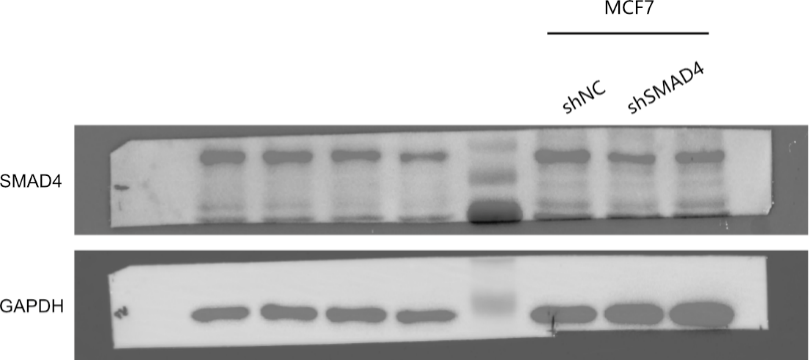


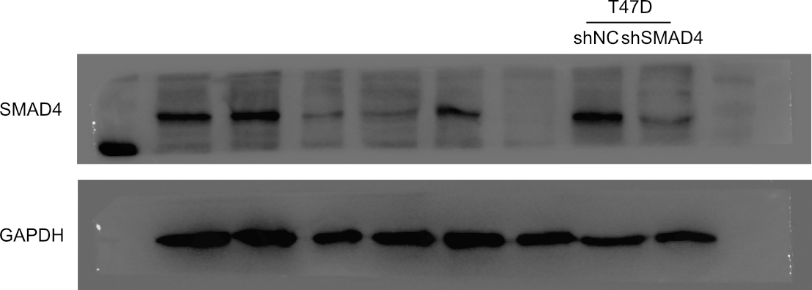


F:


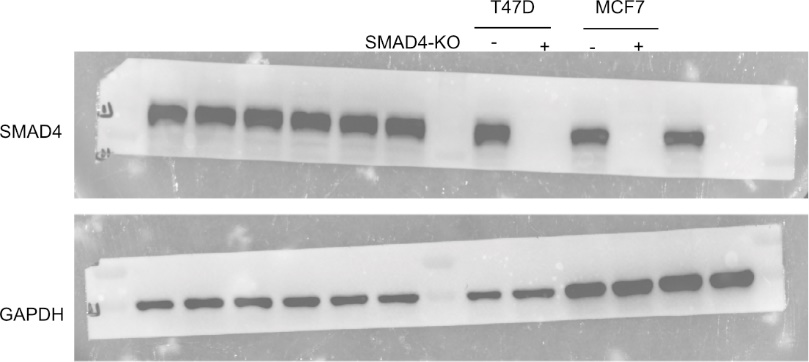


Fig. 3

G, H:


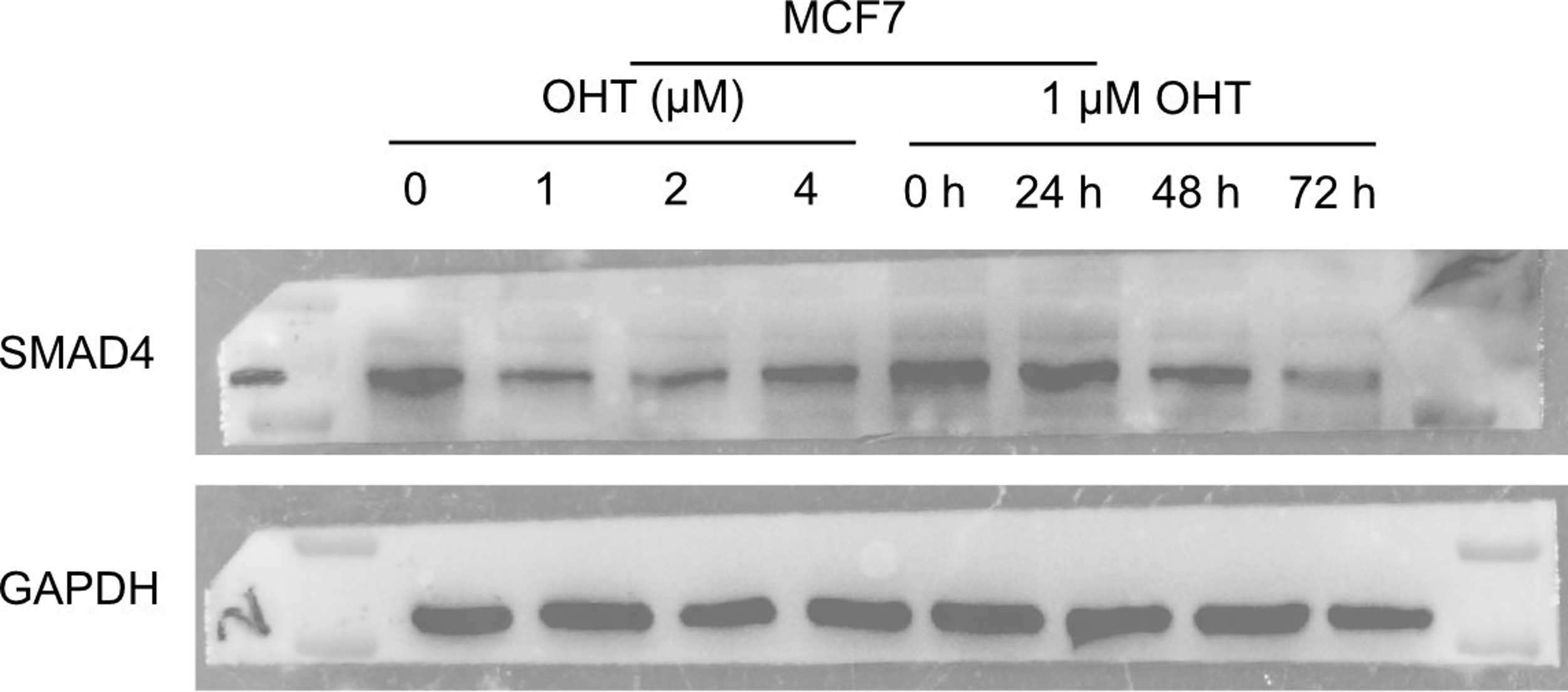


I, L:


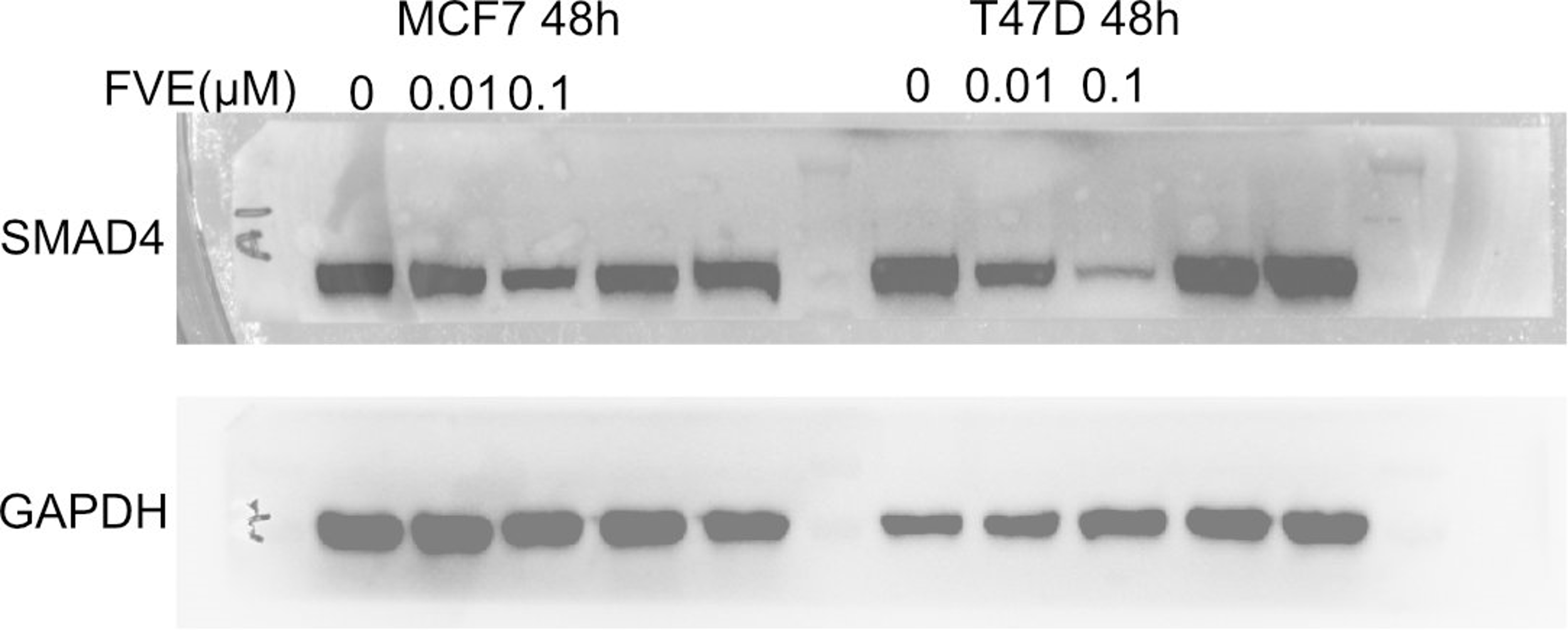


J:


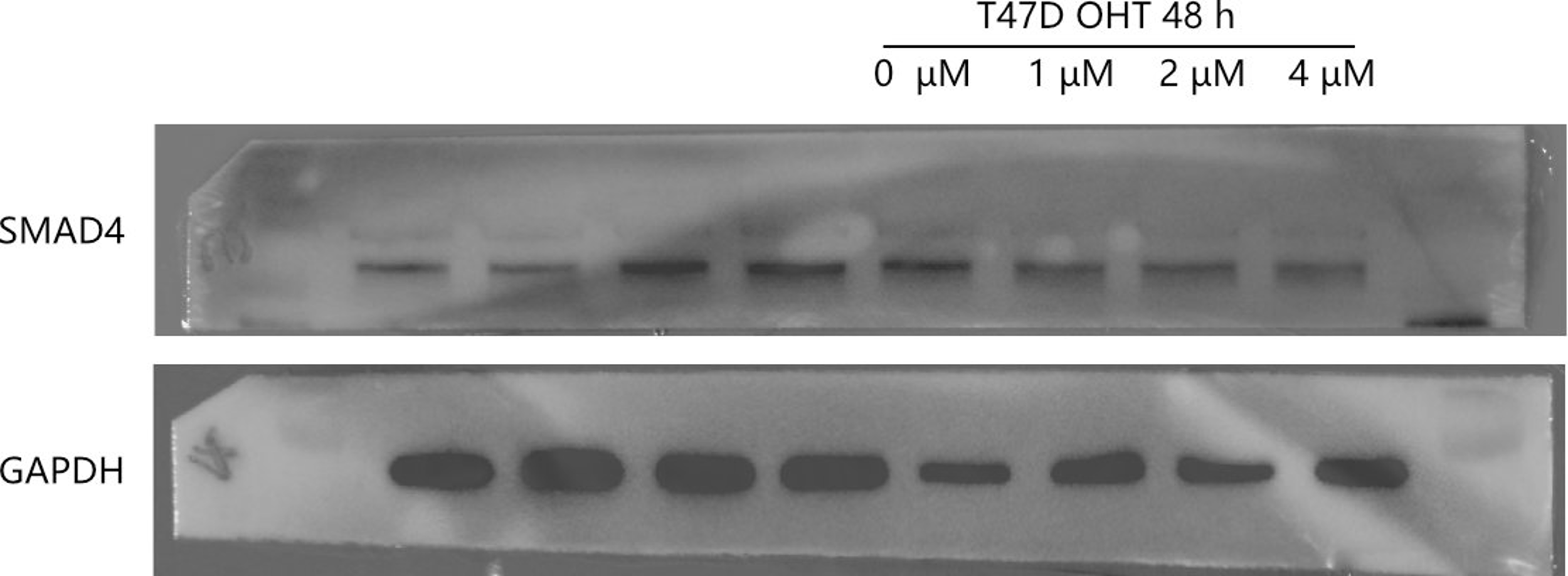


K:


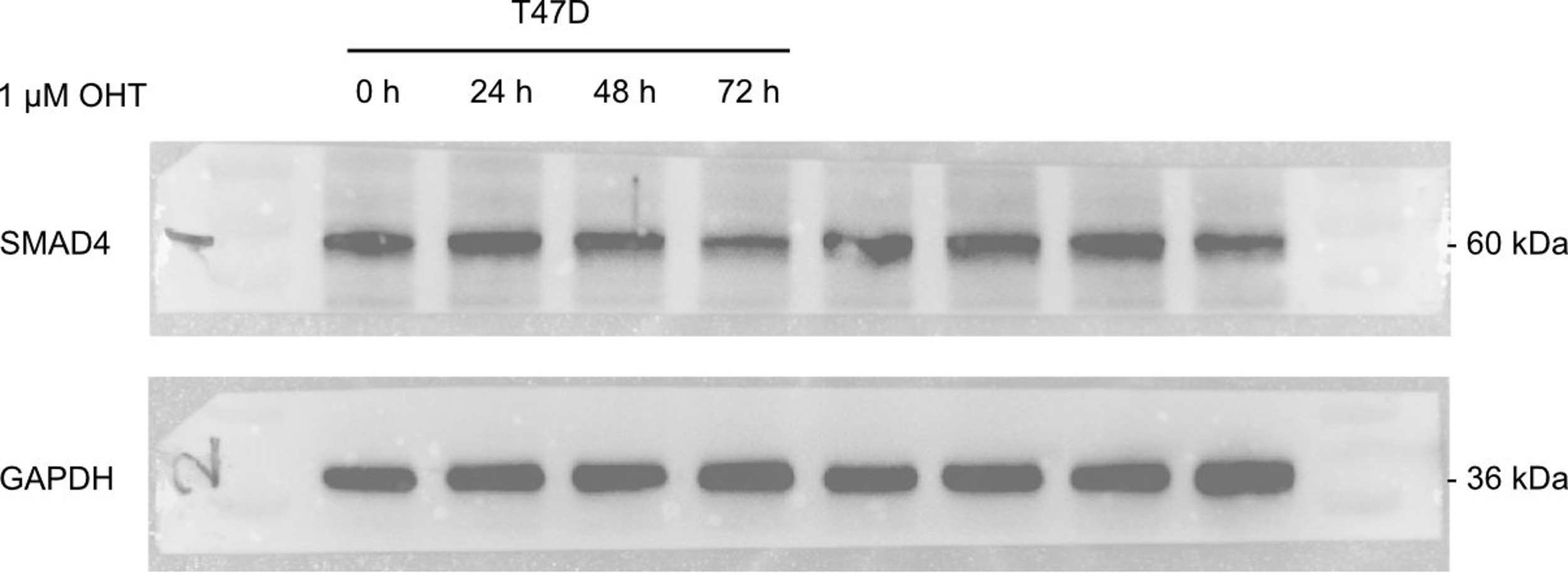


M:


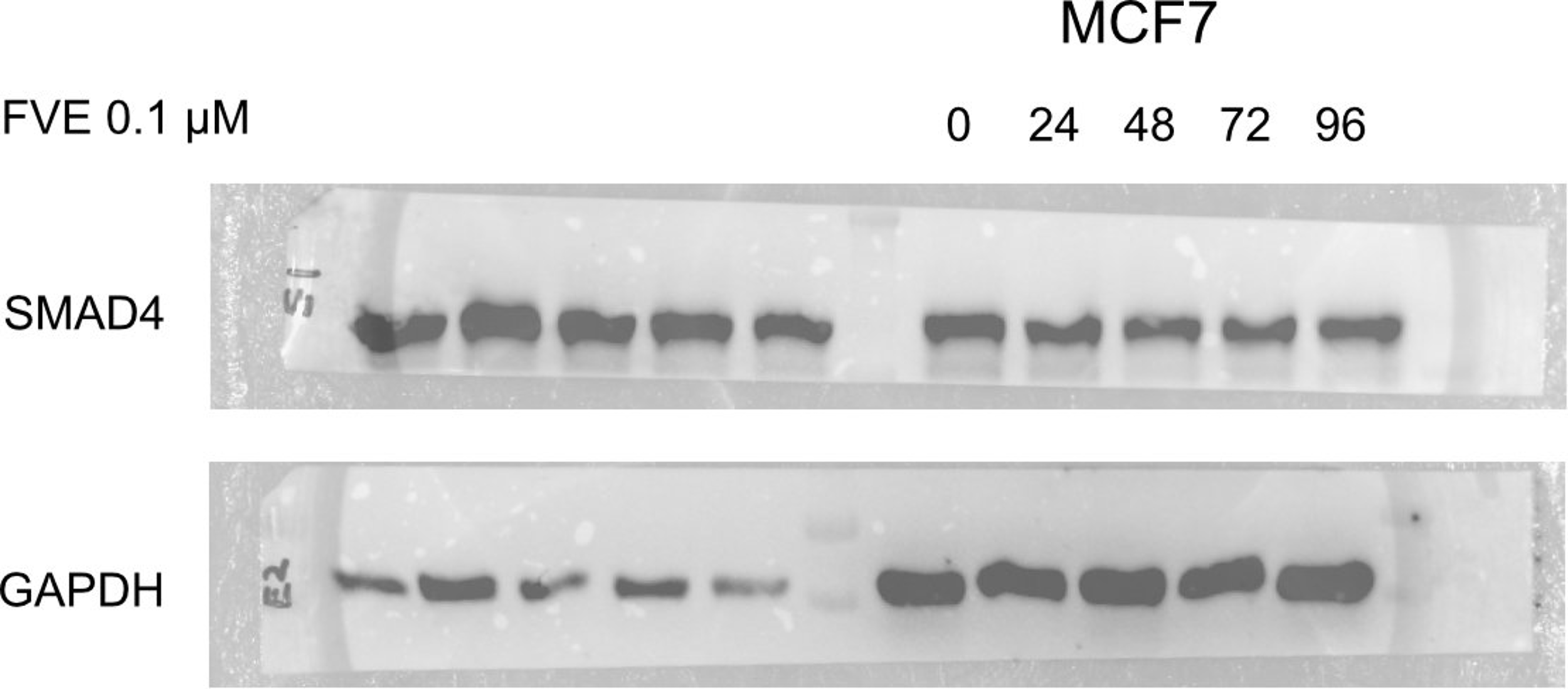


N:


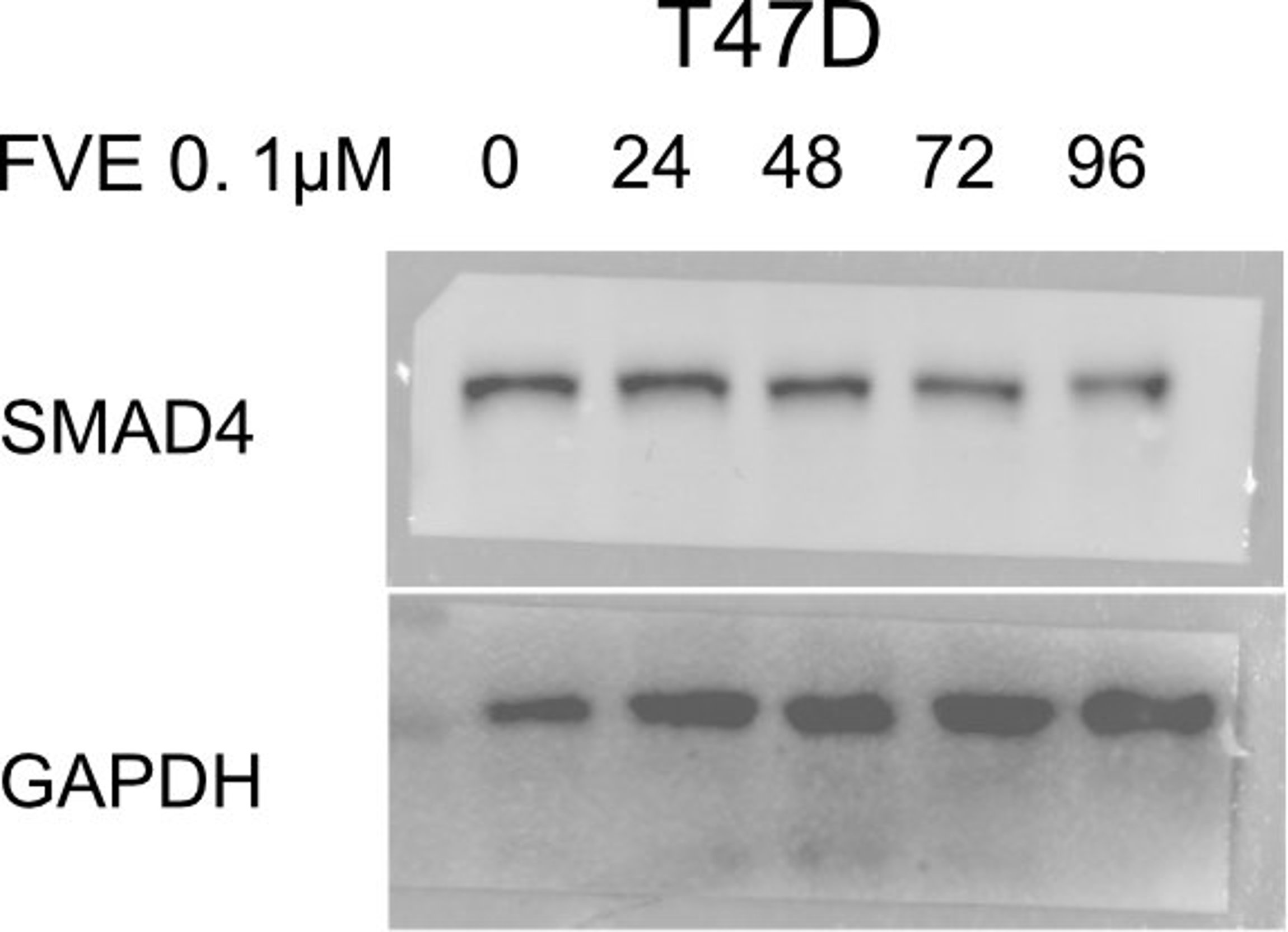


Fig. 5

E:





F:


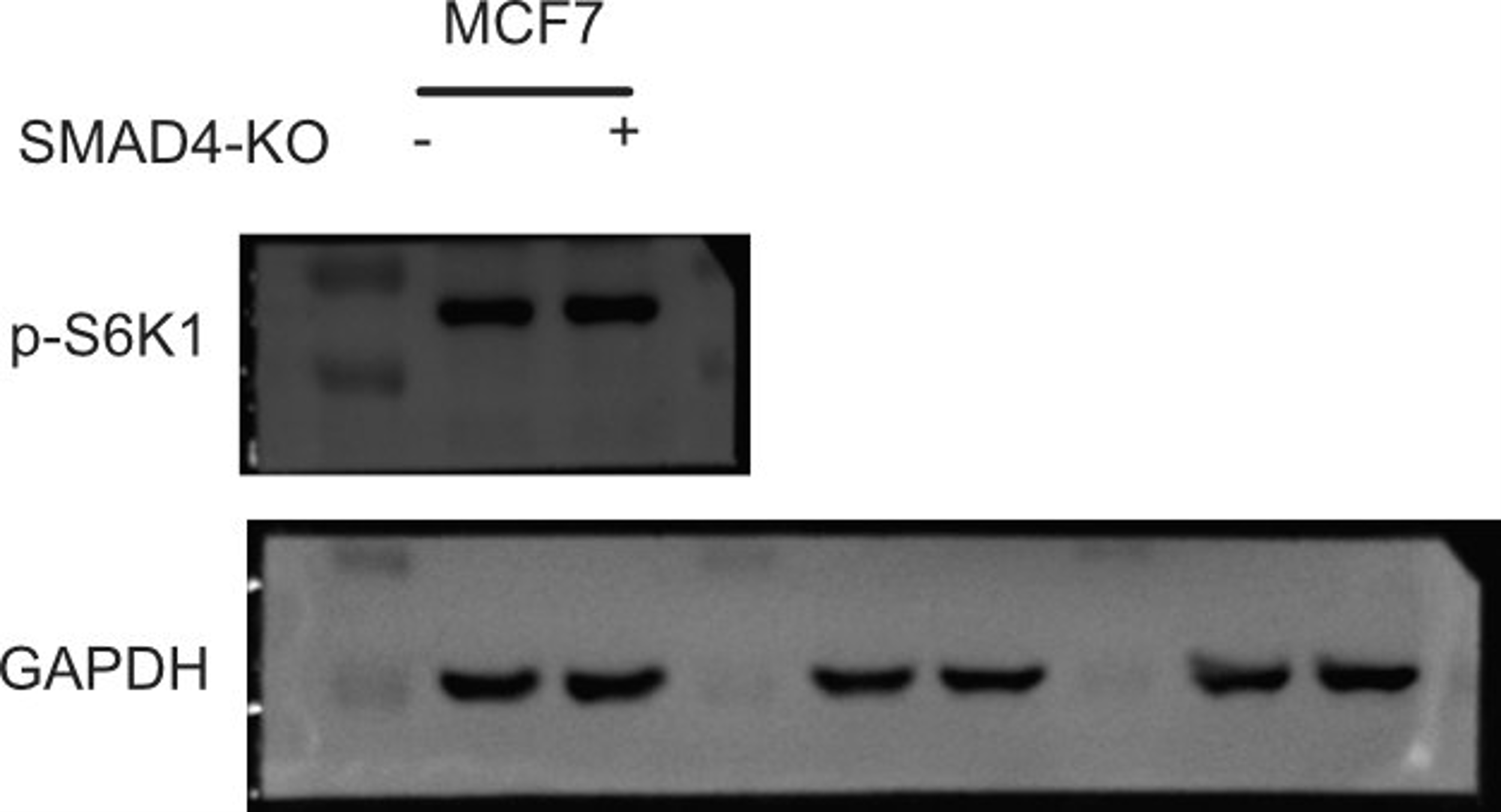


G:


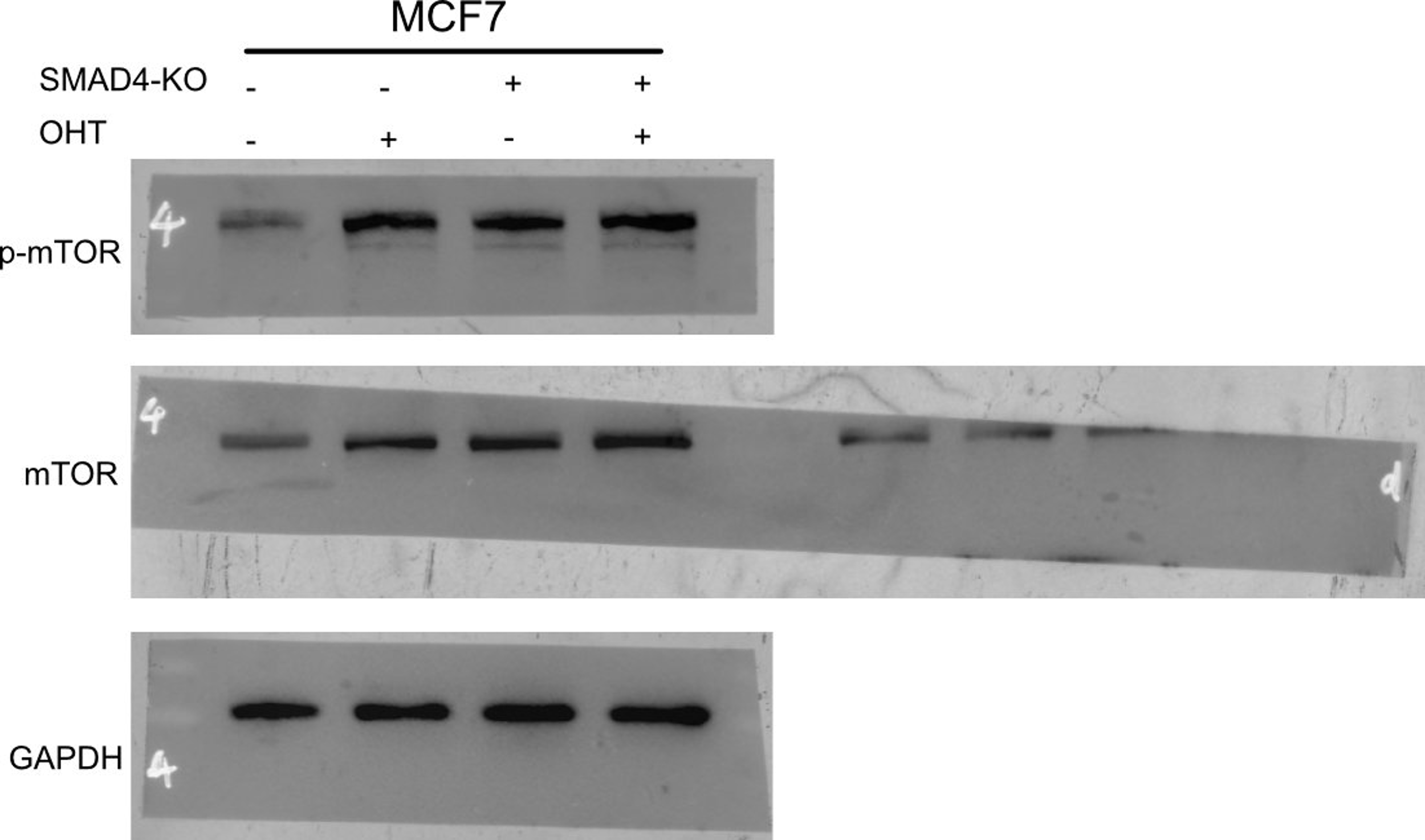


H:


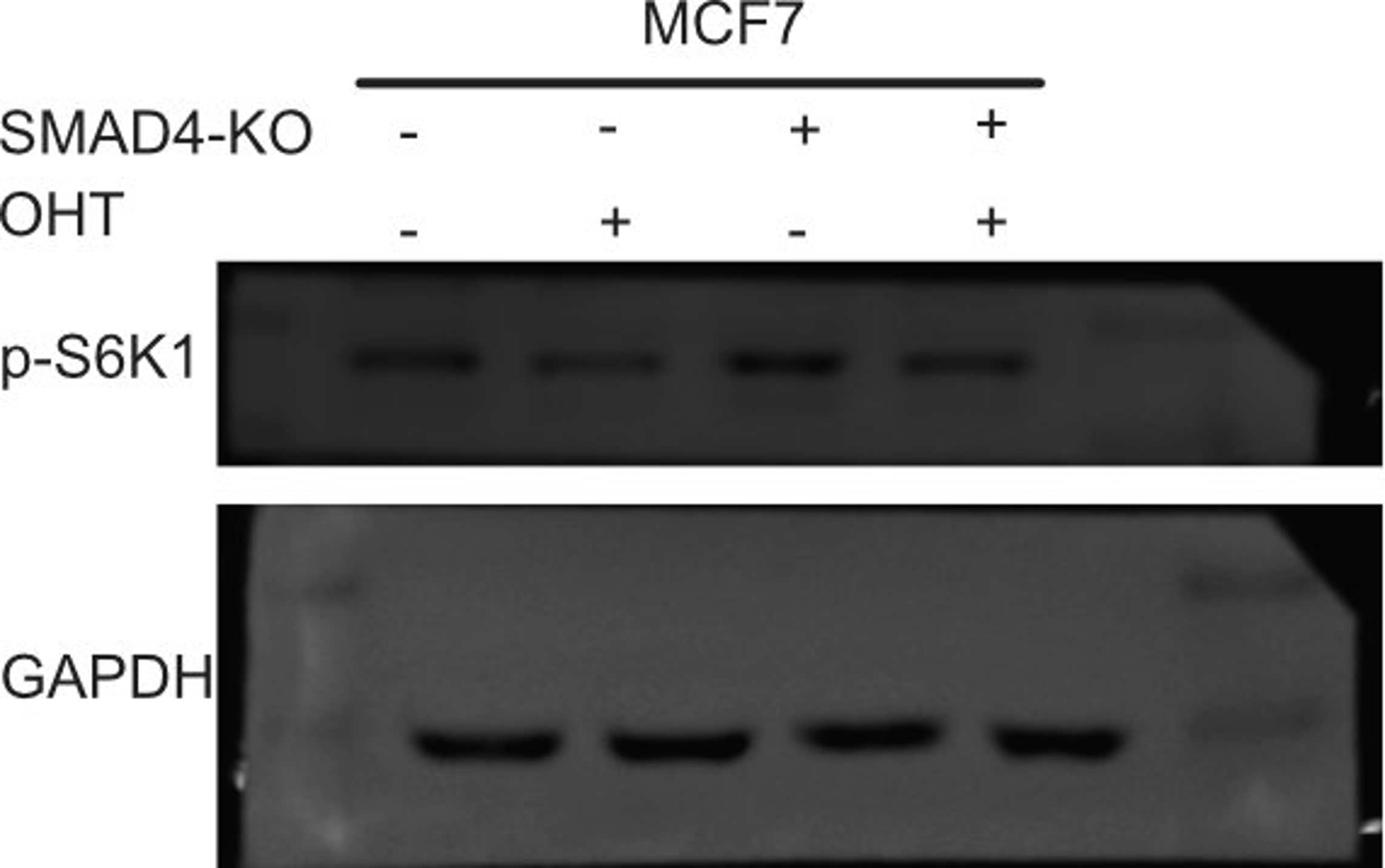


I:


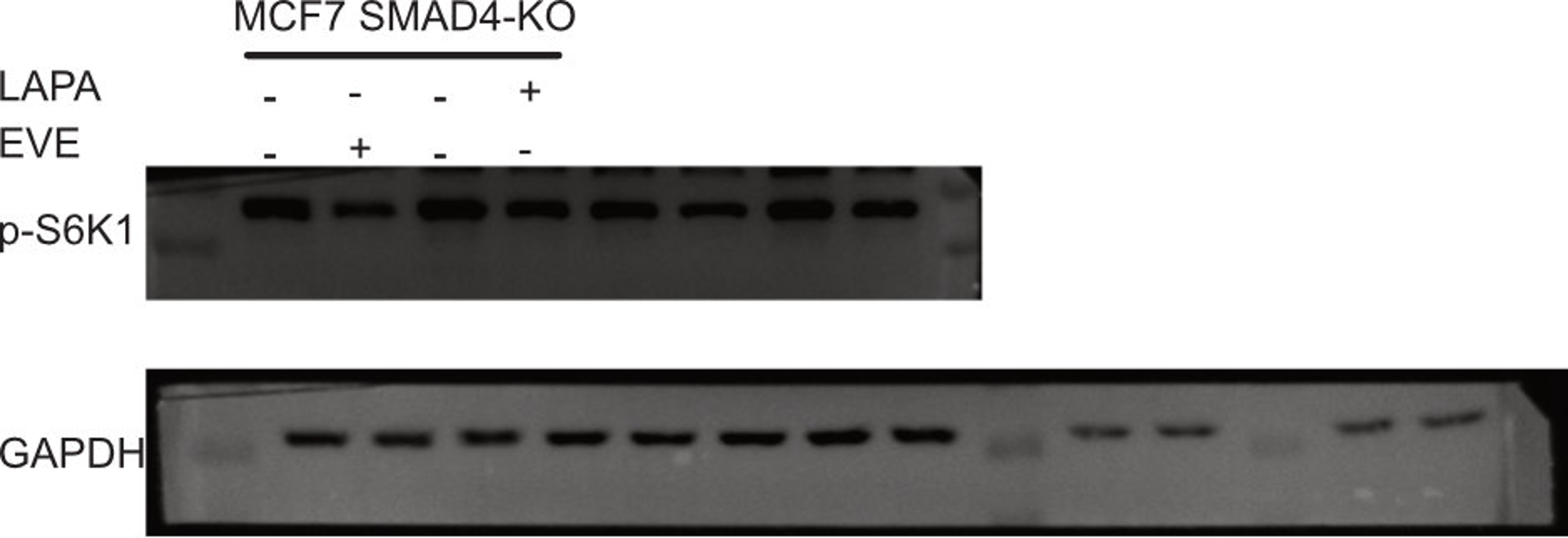


Supplementary Fig. 5

B


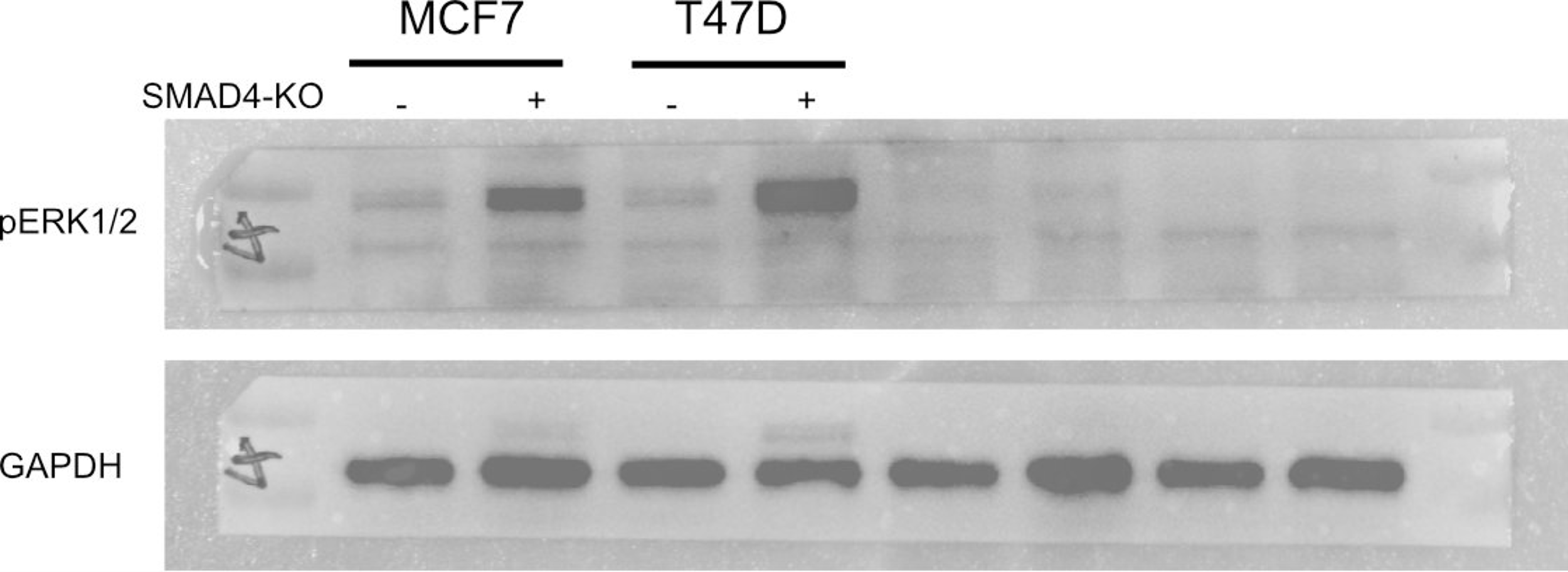


Fig. 6

G:


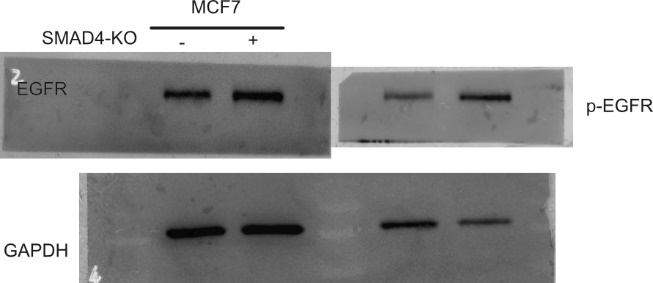


H:


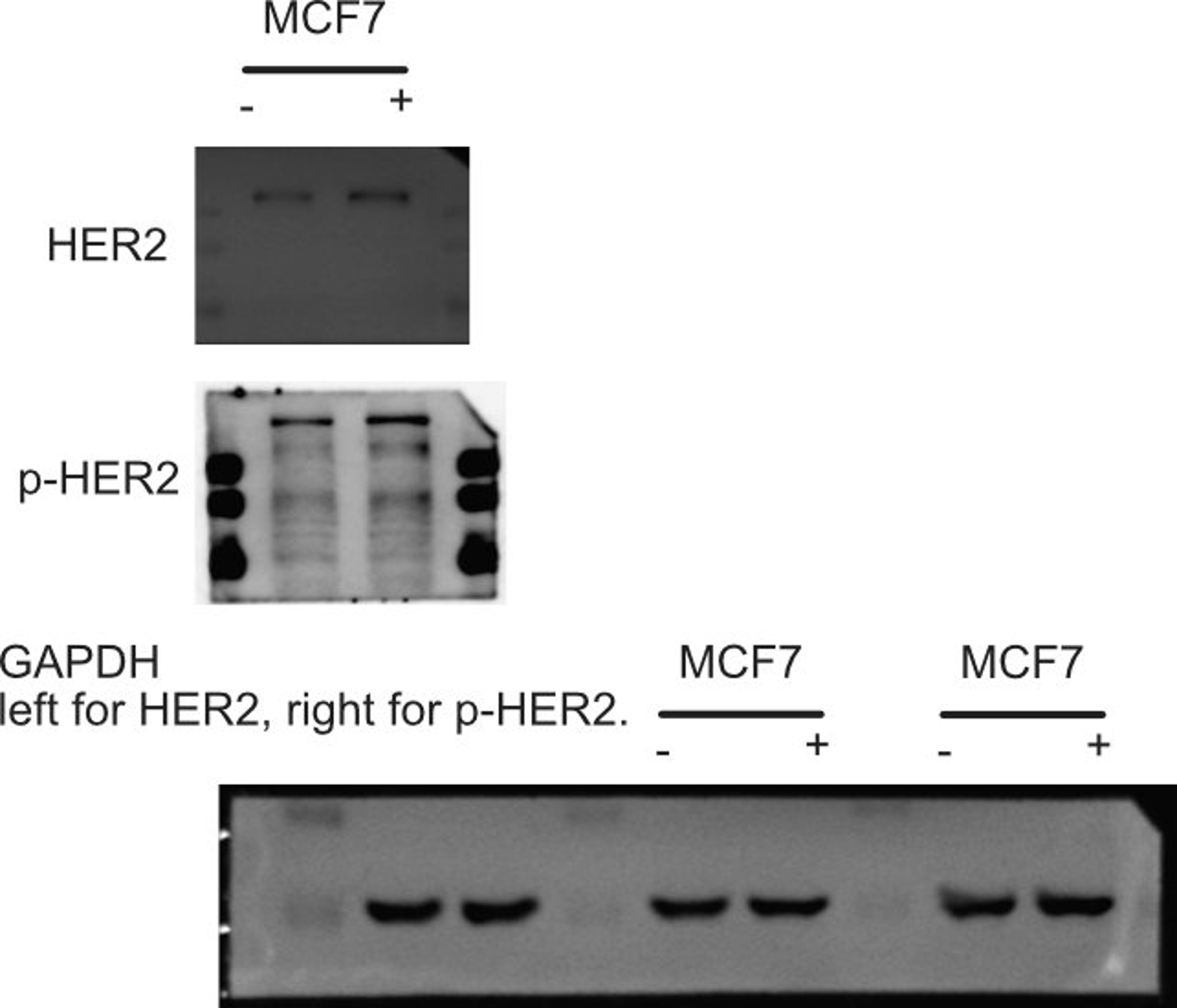


Fig. 7

D:


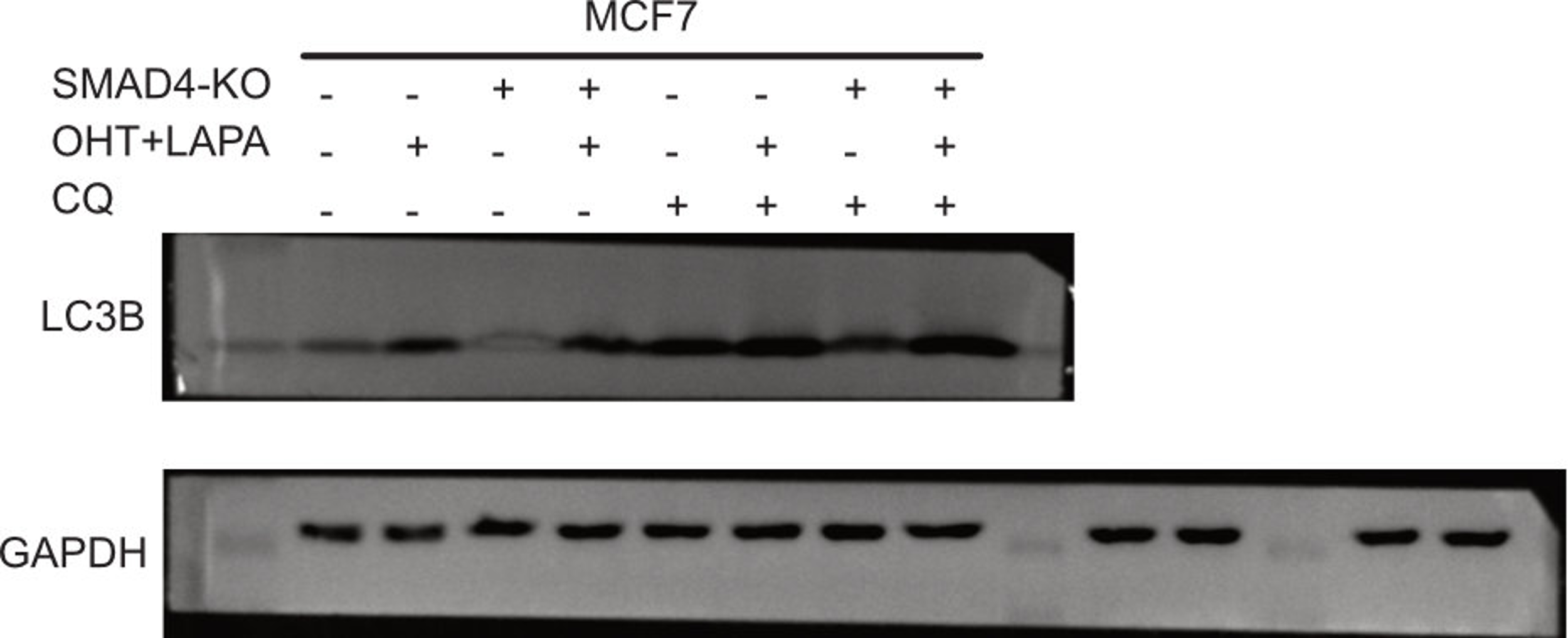


E:


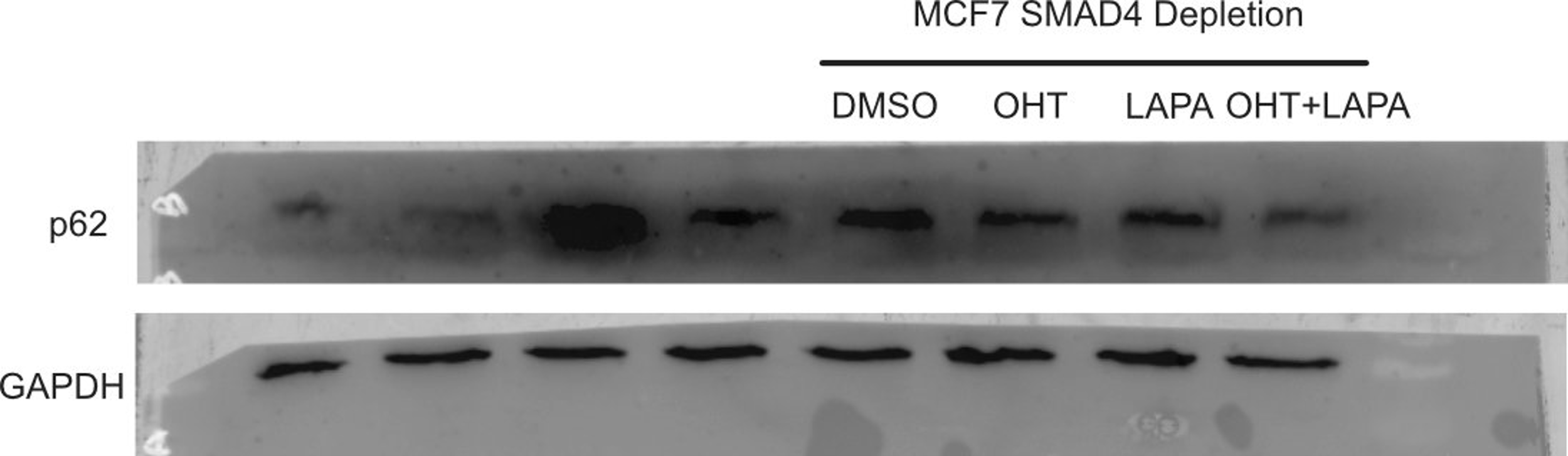


F:


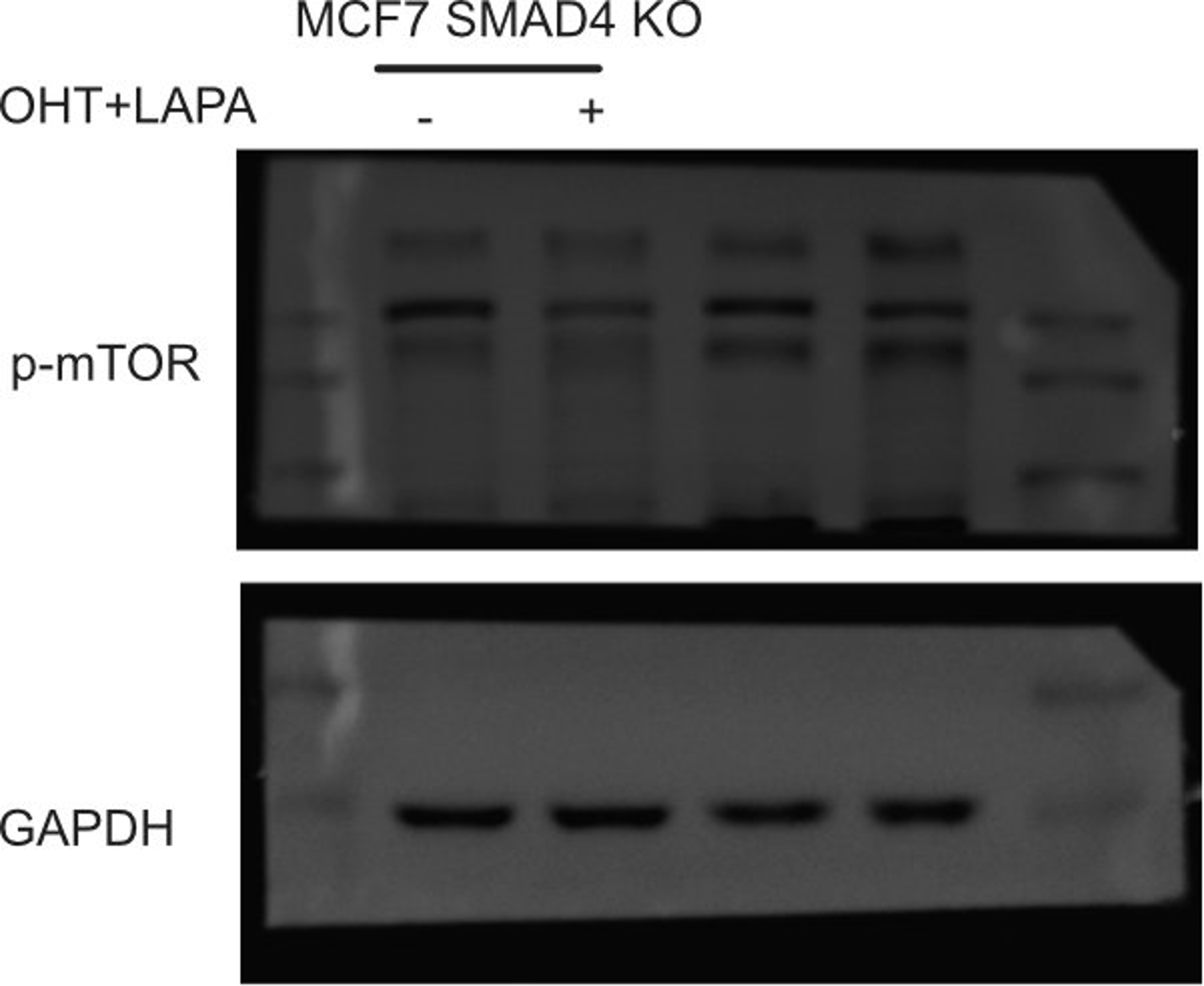

Supplement: Supplementary file 8 — original data [file 41419_2024_6838_MOESM8_ESM.docx]
